# Expanding Configurational Complexity through Dipole Dilution in Pseudohalide Argyrodite Ion Conductors

**DOI:** 10.1021/acs.chemmater.5c02236

**Published:** 2025-12-04

**Authors:** Shelby L. Galinat, Claire M. Willard, Krishna Teja Valeti, Rebecca W. Smaha, Anna Staerz, Annalise E. Maughan

**Affiliations:** † Materials Science Program, 3557 Colorado School of Mines, Golden, Colorado 80401, United States; ‡ Department of Chemistry, Colorado School of Mines, Golden, Colorado 80401, United States; § Department of Metallurgical and Materials Engineering, Colorado School of Mines, Golden, Colorado 80401, United States; ∥ Materials Science Center, 53405 National Renewable Energy Laboratory, Golden, Colorado 80401, United States

## Abstract

The advantageous properties of (pseudo)­halide argyrodite ion conductors of the formula Li_6_PS_5_ *X* (*X* = Cl^–^, Br^–^, I^–^, CN^–^) have motivated extensive studies of their structure-transport relationships, particularly as they pertain to the role of atomic site disorder. The argyrodite structure can accommodate additional configurational complexity to promote ion transport via extended three-anion site mixing and the potential for orientational disorder of molecular anions. In this work, we explore a ternary anion system including the cyanide anion, expanding site disorder and introducing dipolar orientations as an additional degree of freedom. We prepared the series Li_6_PS_5_(CN)_1–*x*_Br_*x*_, in which the dipolar cyanide anions are systematically diluted with bromide. We find that anion disorder, as determined by synchrotron and neutron diffraction and quantified by configurational entropy (*S*_config_), is correlated with lowered activation barriers and increased lithium ion conductivity. We propose that *S*_config_ describes the electrostatic heterogeneity of the Li environments, flattening the energetic landscape for ion transport. While anion substitution strongly impacts the activation barrier for transport, the temperature-independent Arrhenius prefactor does not follow the same trend. Through heat-capacity measurements of attempt frequency and deconvolution of terms within the prefactor, we rationalize the apparent decoupling of activation energy and prefactor to strong cyanide-lithium interactions that increase the entropy of migration. Together, these results expand the structure–property relationships in the argyrodite family to encompass multiple facets of disorder and the subsequent impact on lithium ion transport.

## Graphical abstract

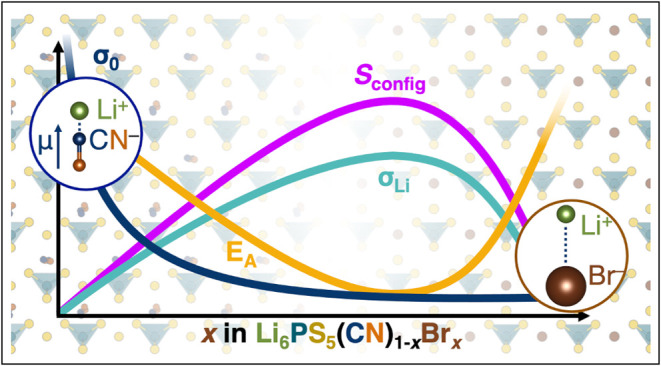

## Introduction

Fast ion transport in the solid state is key to unlocking next-generation energy storage technologies, from fuel cells to solid state batteries. Halide argyrodites of the form Li_6_PS_5_ *X*, where *X* ^–^ is a monovalent anion, can exhibit lithium ion conductivities on the order of 10 mS cm^–1^ at room temperature that make them desirable for potential applications in all-solid-state batteries.^1–4^ The exceptional ionic conductivities of the argyrodite family arise from a combination of favorable structural features. The cubic structure (space group *F*4̅3*m*) is composed of isolated PS_4_^3–^ tetrahedral units that enable three-dimensional ion diffusion. The halide *X* ^–^ anions and additional “free” S^2–^ anions decorate two interpenetrating face-centered cubic lattices originating at the *4a* and *4d* Wyckoff positions, and can exhibit a large degree of disorder across the two sites (Figure 1a).^5–10^ The polarizability of the halide and sulfide anions facilitate low-energy ion transport by screening electrostatic interactions between lithium ions and the host framework and softening the energetic landscape for ion hopping.^6,11^ The lithium cations reside in quasi-octahedral cages surrounding the anion at the 4*d* site and are highly disordered; macroscopic ionic conductivity is mediated by longer-range lithium ion hopping between neighboring cages.^12–15^

**Figure 1:**
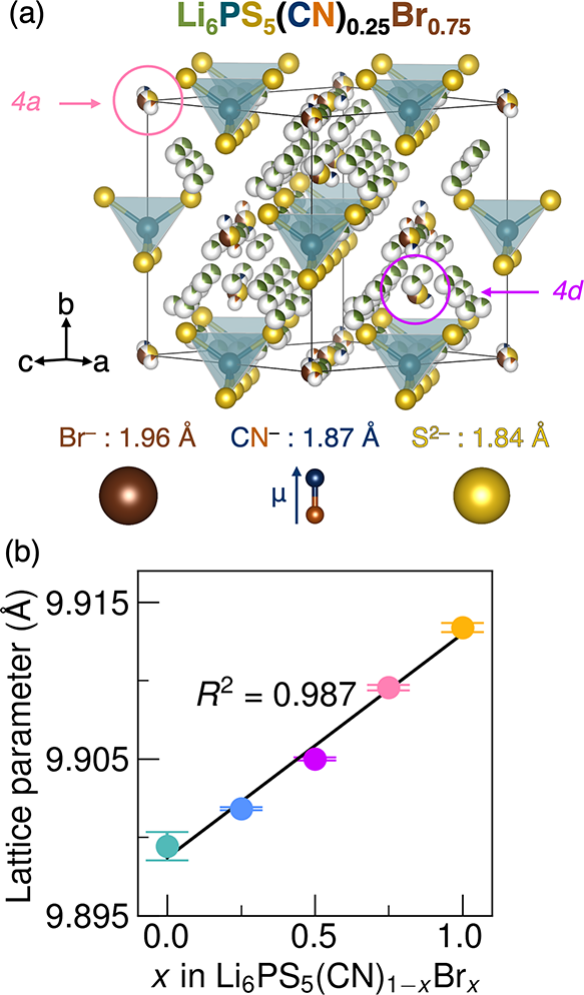
(a) Pseudocubic structural model of Li_6_PS_5_(CN)_0.25_Br_0.75_ displaying orientational disorder of CN^–^ dipoles. Li_6_PS_5_(CN)_1–*x*_Br_*x*_ crystallizes in the *F*4̅3*m* spacegroup. Site disorder between the *4a* and *4d* Wyckoff positions results in partial CN^–^, Br^–^, and S^2–^ occupancy on these sites, depending on the composition. (b) The refined lattice parameters for Li_6_PS_5_(CN)_1–*x*_Br_*x*_ increase linearly with *x*. Error bars represent error from the Rietveld refinement of synchrotron X-ray diffraction of powder samples with Si internal standards (as shown in Figure S5).

Anion disorder strongly impacts the long-range Li^+^ transport processes that contribute to high macroscopic ionic conductivities in the argyrodites. In particular, the presence of site disorder between the S^2–^ and *X* ^–^ anions across the *4a* and *4d* Wyckoff sites lowers the activation barrier for intercage Li^+^ hopping.^5,10,15–17^ This behavior is attributed to averaging of the divalent and monovalent anion charges between the *4a*/*4d* sites that encourages disordering of the lithium substructure.^5,10,14,15,17^ Recent studies have extended the role of site disorder through introduction of additional halide anions in compositionally complex argyrodites.^6,18–21^ Li et al. recently quantified the distribution of S^2–^, Cl^–^, and Br^–^ anions across two sites in Li_5.5_PS_4.5_Cl_*x*_Br_1.5–*x*_ through configurational entropy (*S*_config_) and found that higher ionic conductivities are correlated with increased *S*_config_.^19^ This observation suggests that a broadened definition of site disorder which encompasses isovalent substitution is necessary for understanding how compositional complexity tunes the energetic landscape for ion hopping.

Molecular anions present an opportunity to broaden the extent of disorder in solid state electrolytes. Pseudohalides—such as cyanide (CN^–^), thiocyanate (SCN^–^), borohydride (BH_4_^–^), or tetrafluoroborate (BF_4_^–^)—are molecular anions that mimic the coordination preferences of halides.^22^ The distinct geometries and form factors of molecular anions can imbue additional degrees of disorder beyond their monatomic counterparts. In addition to site mixing across multiple crystallographic sites (*vide supra*), pseudohalides can exhibit orientational disorder about their centers of mass.^23^ The configuration of orientational order is governed by the physical geometry and electrostatics of the coordination environment in which the anions reside, including via dipole–dipole interactions or local strain.^24–26^ Our recent studies of the cyanide argyrodite Li_6_PS_5_CN have found that cyanide exhibits both orientational disorder and site mixing across the 4*a* and 4*d* sites.^27,28^ Furthermore, species with a net dipole moment can act to screen the charge of neighboring ions or participate in dielectric relaxation processes.^29^ Beyond static disorder, thermally activated rotations of these molecular species may also introduce dynamic disorder; coupling of molecular anion rotations and cation migration has been suggested in a variety of materials.^30–37^ Recent studies of LiSCN have shown that SCN^–^ anions undergo reorientations under an applied electric field that appear to be coupled with Li^+^ hopping.^38^ Additional studies are needed to disentangle the relationships between multiple facets of disorder and ion transport in pseudohalide argyrodites.

In order to evaluate the impact of site mixing and orientational disorder on ion migration in pseudohalide argyrodites, we have prepared the substitution series Li_6_PS_5_(CN)_1–*x*_Br_*x*_ (*x* = 0, 0.25, 0.5, 0.75, 1). We find that all members of the series adopt the cubic argyrodite structure, as determined by high-resolution synchrotron and neutron powder diffraction. Room temperature lithium ion conductivity reaches a maximum for *x* = 0.75, concomitant with the lowest activation barrier and maximized configurational entropy (*S*_config_) across the series. As *S*_config_ quantifies the diversity of the local Coulombic interactions caused by ternary anion site mixing, we propose that activation barriers to ion hopping are reduced by the highly disordered anion sublattice. Interestingly, we find that the Arrhenius prefactor does not follow a similar trend with *S*_config_ and does not appear to follow the Meyer-Neldel compensation rule. Through analysis of contributions to the Arrhenius prefactor, we attribute the apparent decoupling of activation barrier and Arrhenius prefactor to the high volumetric charge density of the cyanide anions, which increases the entropy of migration. Building on previous findings that disorder lowers the activation barrier to ion transport, this work also identifies the importance of strong cation-dipole electrostatic interactions on vibrational dynamics and the entropy of migration.

## Experimental Section

### Synthesis

CAUTION. Cyanide salts hydrolyze to form HCN in acidic conditions, which is a highly toxic gas (immediately dangerous to life or health concentration: 50 ppm).^39^ Hydrolysis may also occur in water or on contact with atmospheric moisture. To prevent HCN evolution, all manipulations were performed in moisture-free environments in an argon glovebox or on a Schlenk line. All Schlenk glassware was dried for 12 h at 115 °C and transferred immediately to the glovebox prior to use. All cyanide-containing waste was quenched with bleach.

LiCN was synthesized by adaptation of the method described in a prior report.^40^ First, LiCl (Sigma-Aldrich, anhydrous, 99%) was ball milled at 800 rpm in an MSE PMV1–0.4L planetary ball mill with 5 mm diameter zirconia media in a 50 mL zirconia jar at 15 min on, 15 min off for 4 cycles, reversing directions, and dried under vacuum at 150 °C for 4 h. In an argon glovebox, NaCN (Thermo Scientific, 98%) and the prepared LiCl were hand ground together in an agate mortar and pestle and added to a 100 mL Schlenk flask. Approximately 60 mL anhydrous dimethylformamide (DMF, Sigma-Aldrich, 99.8%) was added via cannula under flowing N_2_, and the suspension was stirred for 4 days. The LiCN-DMF solution was transferred via cannula filter to a 250 mL Schlenk flask, and LiCN-DMF was precipitated out via addition of 60 mL of anhydrous diethyl ether (DEE, Thermo Fisher, ACS reagent). The DEE/DMF solvent was removed by cannula filter, and the precipitate was washed with DEE twice more. The LiCN-DMF precipitate was dried under vacuum at 40 °C for 1 h to remove the DEE. After hand grinding the precipitate in an argon glovebox, 0.6 g of LiCN-DMF were added back to the flask and dried under vacuum at 154 °C for 11 h, melting the LiCN and removing the residual DMF.

Li_6_PS_5_(CN)_1–*x*_Br_*x*_ was synthesized on a 0.0024 mol scale of product following the solution-phase synthesis reported previously.^27,28,41^ 3 mol equiv Li_2_S (MSE Supplies, 99.9%) and 1 mol equiv P_2_S_5_ (Sigma-Aldrich, 99%) were hand ground together in an agate mortar and pestle and loaded into a dry 100 mL Schlenk flask. The flask was connected to the Schlenk line under flowing N_2_ and 20 mL of anhydrous tetrahydrofuran (THF, Sigma-Aldrich, 99.9%) was added. The mixture was stirred under flowing N_2_ for 1.25 h, until the suspension transitioned from light yellow, to white, then back to sunny yellow (Figure S2a). To a second 100 mL flask was added 2 mol equiv Li_2_S and 2 mol equiv Li*X* (*X* = CN^–^, Br^–^). Prior to use, the LiBr precursor was ball milled at 800 rpm in an MSE PMV1–0.4L planetary ball mill with 5 mm diameter zirconia media in a 50 mL zirconia jar at 15 min on, 15 min off for 4 cycles, reversing directions, and dried under vacuum at 150 °C for 4 h. 20 mL anhydrous ethanol (EtOH, Sigma-Aldrich, 99.5%) was added via cannula transfer to the second flask to dissolve the Li_2_S and Li*X* precursors. The EtOH solution was added to the THF flask via cannula transfer, turning the solution emerald green (Figure S2b). After stirring for 20 min, vacuum was applied and the flask was heated to 50, 80 and 100 °C for 30 min each to remove solvent and precipitate out a tan powder. Finally, the flask was heated to 150 °C for 2 h to further dry the solid product.

### High-Resolution Synchrotron Powder X-ray Diffraction

Li_6_PS_5_(CN)_1–*x*_Br_*x*_ was added to quartz capillaries (0.68 mm ID, 0.7 mm OD) and sealed with epoxy in an argon glovebox. Additional samples were mixed with Si (Strem) as an internal standard. Powder diffraction data were collected at the Stanford Synchrotron Radiation Lightsource (SSRL) at SLAC National Accelerator Laboratory on beamline BL2–1 with wavelength λ = 0.73 Å. Rietveld refinements were conducted in TOPAS Pro V6.^42^ To generate the color maps of *R*_wp_ shown in Figures 3 and S3, a list of possible anion occupancies on the *4a* and *4d* sites was generated, maintaining stoichiometry. Calculations of *R*_wp_ were then performed in TOPAS by iterating through this list of constrained domains of fixed anion occupancies. All crystal structure figures were generated using VESTA.^43^

### Neutron Powder Diffraction

Neutron powder diffraction data at *T* = 300 K were collected with a wavelength of λ = 1.53655 Å with no collimator at the HB-2A POWDER beamline at the High Flux Isotope Reactor, Oak Ridge National Laboratory. Approximately 0.5–0.6 g of each composition were loaded in 6 mm diameter vanadium cans in a helium glovebox. Scattering data were collected for 1–2 h for each composition. Rietveld refinements were conducted in TOPAS V6 Pro software.^42^

### Fourier-Transform Infrared Spectroscopy

Powdered samples were diluted in a ∼70:1 KBr:Li_6_PS_5_(CN)_1–*x*_Br_*x*_ (by mass) ratio and added to air-free transmission FT-IR cells with NaCl windows in an argon glovebox (KBr: Acros Organics, 99+%, for spectroscopy, IR grade). The sample compartment was placed under vacuum. 160 replicate spectra were collected in transmission geometry on a Bruker Vertex 70v FT-IR spectrometer with an aperture opening of 6 mm and with a resolution of 4 cm^–1^. A background spectrum of a cell loaded with KBr was collected to perform manual background division.

### Raman Spectroscopy

Powdered samples were loaded onto concave microscope slides and sealed with Kapton tape to prevent air exposure. Spectra were collected using a 1064 nm Thermo Scientific iS50 Raman Spectrometer at a resolution of 1 cm^–1^ at 0.1, 0.15, 0.3, 0.25, and 0.05 W laser power for *x* = 0, 0.25, 0.5, 0.75, and 1, respectively. For 0 ≤ *x* ≤ 0.75, 50 replicate spectra were collected with an aperture of 75. For *x* = 1, 100 replicate spectra were collected with an aperture of 10. A dark spectrum was collected with the laser off to perform manual background subtraction.

### AC Electrochemical Impedance Spectroscopy

Inside an argon-filled glovebox, a 6 mm diameter pellet of ∼0.1 g of Li_6_PS_5_(CN)_1–*x*_Br_*x*_ was pressed in a uniaxial pellet press to ∼ 60 bar on the gauge, which is equivalent to 8000 bar pressure over the area of the pellet. Pressing resulted in a pellet ∼2 mm thick with ∼ 80% theoretical density. The pellet was loaded into a custom-built, air-free, insulating polyether ether ketone (PEEK) cell in parallel plate capacitor geometry with 6 mm diameter graphite foil blocking electrodes in contact with stainless steel rods. Measurements were performed under 31.5 MPa of constant pressure as measured by an in-line load cell in a custom pressure jig. EIS measurements were collected using a Gamry Interface 1010E Potentiostat with an applied bias of 20 mV. The frequency was swept logarithmically from 2 × 10^6^ Hz to 0.2 Hz at 10 measurements per decade. The PEEK cell, pressure jig, and load cell assembly were placed in a Quincy Lab convection oven for temperature-dependent measurements at 30 °C and from 35–95 °C in 10 °C increments. EIS measurements were collected continuously during temperature ramping and stabilization. Data sets collected after 2.5 h of thermal equilibration during the stable target temperature window were selected for analysis. EIS data were fit with an (*R*_1_ *Q*_1_) – *Q*_2_ equivalent circuit using *ad hoc* Python code to extract parameters descriptive of the impedance.

### Heat Capacity

Heat capacity measurements were conducted on pelletized samples from *T* = 2–250 K using a Quantum Design Physical Property Measurement System (PPMS). An addenda measurement was performed with Apiezon-N grease for each sample and was subtracted from the total heat capacity. A small pellet (mass ∼ 2–5 mg) was affixed to the Apiezon-N grease on the sample puck. For each measurement, a quasi-adiabatic heat pulse corresponding to a 2% increase in temperature was used and equilibrated for two time constants. Three measurements were collected at each temperature. The first measurement was discarded, and the final two were averaged for each temperature. The low temperature (2 to 10 K) heat capacity was fit using the Debye model to extract the Debye temperature.

## Results and Discussion

### Structure

All members of the Li_6_PS_5_(CN)_1–*x*_Br_*x*_ (*x* = 0, 0.25, 0.5, 0.75, 1) series were synthesized by the low-temperature, solution-phase method that was previously developed for Li_6_PS_5_CN.^27,41^ Observation of the nitrile stretch in transmission Fourier-transform infrared spectroscopy (FTIR) demonstrates successful incorporation of the cyanide anion in all compositions *x* < 1 (Figure S7). Additionally, we observe vibrational modes in Raman spectroscopy that are characteristic of the thiophosphate tetrahedra in the argyrodite structure (Figure S8). All compositions adopt the cubic argyrodite structure (space group *F*4̅3*m*) at *T* = 300 K (Figures 1a and S1), as evidenced by synchrotron powder X-ray diffraction (SXRD) data collected on beamline BL2–1 at the Stanford Synchrotron Radiation Lightsource (SSRL), shown in Figure 2a. We observe a systematic shift of the reflections to lower *Q* with increasing *x* (Figure 2c), which is consistent with an increase in lattice parameters due to substitution of the larger Br^–^ anion (*r*_Br^–^_ = 1.96 Å, *r*_CN^–^_ = 1.87 Å).^44,45^ As shown in Figure 1b, the cubic lattice parameter increases linearly across the series according to Vegard’s law, indicating the formation of a single-phase solid solution. In order to ensure the accuracy of the refined lattice parameters of the series, we also performed Rietveld refinements for samples containing an internal silicon standard (Figure S5). Rietveld analysis with and without the Si standard produce nearly equivalent lattice parameters with small error bars. Increasing Br^–^ concentration is also accompanied by a decrease in the intensity of the 111 reflection at *Q* ∼ 1.1 Å^–1^ relative to the 200 reflection at *Q* ∼ 1.3 Å^–1^ (Figure 2b). As these planes directly intersect the *4a* and *4d* Wyckoff sites on which CN^–^ and Br^–^ reside, the continuous change in these intensities is indicative of systematic changes to the electron density at these sites. For *x* > 0, we observe a minor LiBr impurity, which is commonly observed in both solution and solid-state synthesis methods for Li_6_PS_5_Br.^41,46^ While no other crystalline impurity phases are observed, the diffuse scattering observed at *Q* ∼ 1.5 Å^–1^ may indicate the presence of amorphous or paracrystalline phases that would account for mass balance of phosphorus and sulfur. The P_2_S_6_^4–^ vibrational modes in the Raman spectra (Figure S8) could contribute to this diffuse scattering. Alternatively, trace water contamination in EtOH may degrade the Li_2_S and P_2_S_5_ precursors, resulting in an excess of LiBr.^47^

**Figure 2:**
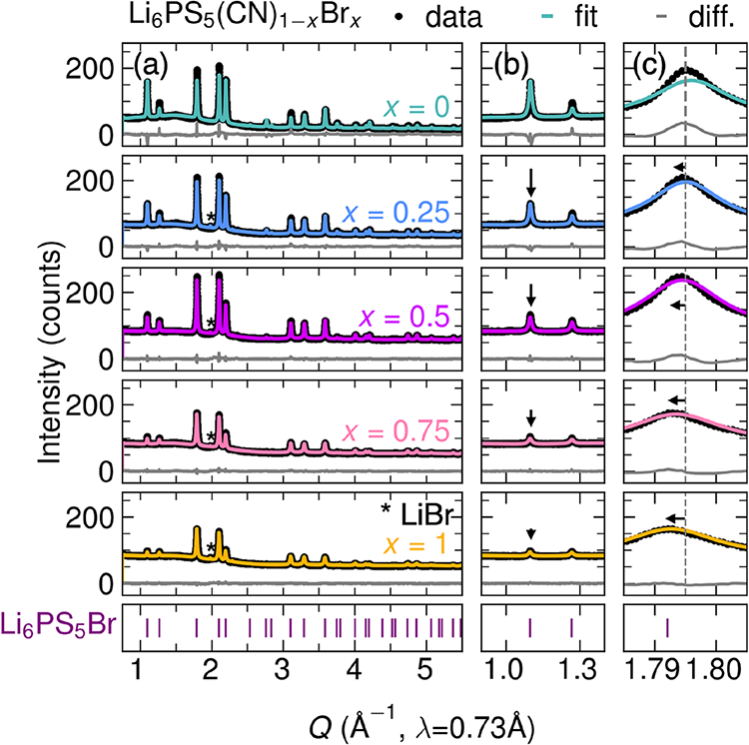
(a) Rietveld refinements of the cubic argyrodite structure (space group *F*4̅3*m*) with room temperature synchrotron powder X-ray diffraction (SXRD) data for Li_6_PS_5_(CN)_1–*x*_Br_*x*_ (*x* = 0, 0.25, 0.5, 0.75, 1) collected on beamline BL2–1 at the Stanford Synchrotron Radiation Lightsource (SSRL) at SLAC National Accelerator Laboratory. Data are shown as black circles, fits are shown as colored lines, and difference curves are shown as gray lines. The purple tick marks show the positions of expected reflections for the cubic argyrodite structure. A minor LiBr impurity is denoted by the asterisk. (b) The intensity of the 111 reflection decreases monotonically relative to the intensity of the 200 reflection with increasing Br^–^ content. (c) We observe systematic peak shifting to lower *Q* with increasing *x*. The gray dashed line is a guide to the eye to highlight peak shifting relative to Li_6_PS_5_CN.

In order to determine how anion substitution impacts the crystal structures along the Li_6_PS_5_(CN)_1–*x*_Br_*x*_ series, we constructed unit cell models for Rietveld refinement. Our previous work suggests that the cyanide anion exhibits orientational disorder in Li_6_PS_5_CN at room temperature.^27,28^ To reflect this orientational disorder, we first centered the CN^–^ bond on the *4a* and *4d* sites, with exact atomic positions constrained as functions of the lattice parameter to preserve the CN^–^ bond length of 1.16 Å.^48^ We aligned the CN^–^ molecules along the [100] and [111] directions for the *4a* and *4d* sites, respectively, in order to intersect the bond axis with the symmetry operations of the *F*4̅3*m* space group. These constraints place the C and N atoms on the *24f* and *16e* Wyckoff positions. The additional C and N positions generated by symmetry capture distinct orientations of the cyanide dipole. The greater multiplicity of these sites is accounted for with proportionally reduced occupancies relative to the 4*a* and 4*d* sites.

Additionally, our unit cell structural models accommodate the potential for different degrees of site disorder of the sulfide, cyanide, and bromide anions across the *4a* and *4d* Wyckoff sites. As both Li_6_PS_5_Br and Li_6_PS_5_CN exhibit site disorder between (pseudo)­halide/sulfide sublattices, we also expect that the Li_6_PS_5_(CN)_1–*x*_Br_*x*_ series will exhibit site mixing among all three anions across these two crystallographic positions.^27,28^ In order to maintain stoichiometry, the occupancies of the free S^2–^, CN^–^, and Br^–^ sites were constrained to sum to 1 S^2–^, (1 – *x*) CN^–^ molecule, and *x* Br^–^. Following the Li_6_PS_5_ *X* argyrodite structure, we also constrained the total occupancy on the *4a* and *4d* sites to 1 anion. As the strongest X-ray scatterer, Br^–^ occupancy was refined first. This value determined the minimum and maximum occupancy values for S^2–^ to maintain stoichiometry. Lastly, the CN^–^ occupancy was fixed to the residual occupancy on the site (1–Br^–^–S^2–^). For each species, the *4a* site occupancy was refined first, and the *4d* occupancy was fixed to the residual stoichiometry.

We also attempted to refine the lithium positions against the SXRD data. However, the weak X-ray scattering from Li precluded refinement of positions, occupancies, and thermal parameters. For our unit cell model, we used the Li positions as refined from neutron diffraction data by Minafra et al. and fixed the atomic displacement parameters (ADPs) to 0.05 Å^2^.^14^

Rietveld refinements against the SXRD data indicate that sulfide/(pseudo)­halide site disorder persists across the solid solution. There is some S^2–^, CN^–^, and Br^–^ occupation on the 4*a* site across all compositions (excluding CN^–^ and Br^–^ for *x* = 1 and *x* = 0, respectively). Sulfur prefers the 4*a* site in Li_6_PS_5_CN with an occupancy of 0.62(1), but prefers the 4*d* site in all other compositions. This is in good agreement with our previous studies of Li_6_PS_5_CN which show 0.62(1) and 0.67(9) S^2–^ on 4*a*.^27,28^ In Li_6_PS_5_Br, Br^–^ exhibits near equal site mixing between the *4a* and *4d* sites (0.508(3) Br^–^ on *4a*). We previously refined the Br^–^ occupancy on 4*a* to 0.551(2), and Yubuchi et al. found a similar value of 0.523(6) for their solution phase synthesis.^27,49^ In contrast, Li_6_PS_5_Br prepared by solid-state synthesis generally exhibits much lower site mixing (around 80% Br^–^ occupancy on 4*a*).^46^ Atomic displacement parameters (ADPs) were also refined for the P and S atoms in the PS_4_^3–^ units and are included in Table S1. Refining the ADPs for the anions across the 4*a* and 4*d* sites proved to be challenging due to the presence of extensive site mixing coupled with orientational disorder of the cyanide anions. This is reflected in the strong correlation between occupancies and ADPs (>85%). As such, we elected to fix the ADPs for these atoms in our final refinements. The structural parameters determined from Rietveld refinements of the constrained models are summarized in Table 1.

**Table 1:** Lattice Parameters, Site Occupancies, and *R*_wp_ Values Determined from Rietveld Refinement of Synchrotron Powder X-ray Diffraction of Li_6_PS_5_(CN)_1–*x*_Br_*x*_ (*x* = 0, 0.25, 0.5, 0.75, 1)^a^

*x*	*a* (Å)	S 4a	CN 24f	Br 4a	S 4d	CN 16e	Br 4d	*R*_wp_ (%)
0	9.8994(9)	0.62(1)	0.063(2)	–	0.38(1)	0.155(3)	–	5.59
0.25	9.9018(1)	0.054(3)	0.116(5)	0.250(7)	0.95(3)	0.014(8)	0 × 10^–3^(7)	2.16
0.5	9.9050(1)	0.14(4)	0.083(6)	0.357(8)	0.86(4)	0 × 10^–3^(9)	0.143(8)	1.55
0.75	9.9095(2)	0.48(6)	0.02(1)	0.39(1)	0.52(6)	0.03(2)	0.36(1)	1.21
1	9.9134(3)	0.492(3)	–	0.508(3)	0.508(3)	–	0.492(3)	1.18

a Additional refined parameters are included in Table S1.

Although there are many possible permutations of anion site disorder in Li_6_PS_5_(CN)_1–*x*_Br_*x*_, our analysis confirms that Rietveld refinement converges to the model with the best fit to the data. In order to better quantify our confidence in the refined occupancies, we systematically calculated the *R*_wp_ for the full range of possible S^2–^/Br^–^/CN^–^ occupancy combinations across the Li_6_PS_5_(CN)_1–*x*_Br_*x*_ series. Color maps of *R*_wp_ as a function of fixed occupancy in Figure 3 indicate that there are a range of possible sulfide and (pseudo)­halide occupancies which produce similar fits to the data. The best refined occupancy values, denoted by the black circles (Table 1), reside in the region of lowest *R*_wp_ in all color maps, which indicates that the refined values represent the true minimum of the least-squares refinement. However, the large range of combinations that result in similar fit statistics illustrates the nuance of structure solution for these heavily disordered materials. Analogous plots for the end-members are shown in Figure S3.

**Figure 3:**
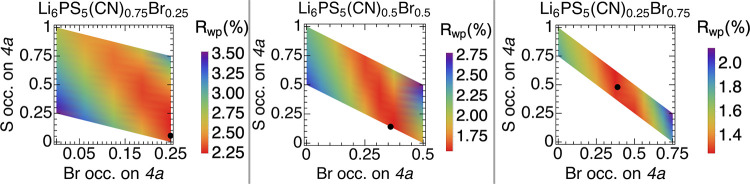
*R*_wp_ color maps for S^2–^/X^–^ (X = CN^–^, Br^–^) fixed occupancies on the 4*a* site for the mixed compositions of Li_6_PS_5_(CN)_1–*x*_Br_*x*_ (0.25 ≤ *x* ≤ 0.75). The black circles represent freely refined values. Wide ranges of S^2–^ and Br^–^ occupancies are possible, with limits on these occupancies constrained to ensure total occupancy on each site sums to 1. Analogous plots for the end-members are shown in Figure S3.

Precise refinement of CN^–^ positions, occupancies, and thermal parameters is challenging due to the relatively low X-ray scattering strength of C and N and the distribution of the atoms over a large volume due to orientational disorder. Our previous work suggests that CN^–^ exhibits static orientational disorder in Li_6_PS_5_CN at room temperature.^28^ While static vs dynamic orientational disorder of CN^–^ is indistinguishable in diffraction, preferred orientations of CN^–^ will reduce the symmetry of the crystal structure and result in the emergence of additional reflections. For example, antiferroelectric ordering of CN^–^ has been observed in neutron diffraction via symmetry reduction from cubic to orthorhombic crystal systems in KCN and NaCN.^50^ In this light, we attempted to use rigid body modeling to refine CN^–^ orientations in a pseudocubic 3 × 3 × 3 supercell of Li_6_PS_5_CN (*P*1 symmetry). Using simulated annealing, the cyanide anions were permitted to rotate independently as rigid bodies about their centers of mass. However, we found that the resulting models did not improve the fit to the SXRD data relative to the cubic *F*4̅3*m* unit cell model with orientationally disordered CN^–^ anions (*R*_wp_ = 6.13% for the supercell, *R*_wp_ = 5.59% for the cubic model). We note that the simulated X-ray diffraction pattern for a unit cell model of ferroelectric CN^–^ exhibits only very subtle intensity differences relative to the *F*4̅3*m* unit cell model with orientationally disordered CN^–^ anions (Figure S4). As changes to the diffraction pattern are nearly indistinguishable even in the fully ordered example, we surmise that the X-ray diffraction data does not provide sufficient sensitivity to resolve domains of locally ordered CN^–^ orientations.

Room temperature neutron powder diffraction (NPD) collected at the High Flux Isotope Reactor (HFIR) at Oak Ridge National Laboratory (ORNL) confirms the cubic argyrodite structure across *x* (Figure S6). The refined lattice parameters for each composition are in reasonable agreement (within ∼ 0.01 Å with the SXRD data (Table S3)). However, refined S^2–^, Br^–^, and CN^–^ occupancies on the *4a* and *4d* Wyckoff positions do not agree well with SXRD refinements (Table S2). We suspect that this deviation arises from poor signal-to-noise in our NPD patterns, due to a combination of the large absorption cross-section of ^6^Li, the low coherent scattering cross section of sulfur, and the large incoherent background from potential hydrogen contamination. Although neutron scattering can provide improved sensitivity to Li, C, and N compared to X-rays, attempted refinements of the Li (Table S3) and CN species did not notably improve the fits to the data. We therefore place more confidence in the SXRD refinements, particularly for high *Z* species such as S^2–^ and Br^–^.

### Ion Transport

In order to determine the lithium-ion transport behavior of the Li_6_PS_5_(CN)_1–*x*_Br_*x*_ series, we performed AC electrochemical impedance spectroscopy (EIS). Symmetric cells were assembled in a parallel-plate capacitor geometry with blocking graphite foil electrodes. Representative Nyquist plots of the real and imaginary contributions to the impedance for all compositions at room temperature are shown in Figure 4a. Across all compositions, the room temperature data are well modeled with an (*R*_1_ *Q*_1_) + *Q*_2_ equivalent circuit. We assign the high-frequency (*R*_1_ *Q*_1_) element to bulk ion transport, which is supported by capacitance values on the order of 1 × 10^–12^ F (Tables S5–S14).^51^ The final constant phase element (*Q*_2_) models the low-frequency capacitive feature due to the graphite blocking electrodes. A constant phase element was chosen instead of a capacitor to account for imperfect interfaces between the electrolyte pellets and the graphite foil. Bulk ionic conductivity σ was calculated from σ = *t*/(*RA*), where *t* and *A* are the thickness and cross-sectional area of the pellet, respectively, and *R* is the resistance from the (*R*_1_ *Q*_1_) circuit element.

**Figure 4:**
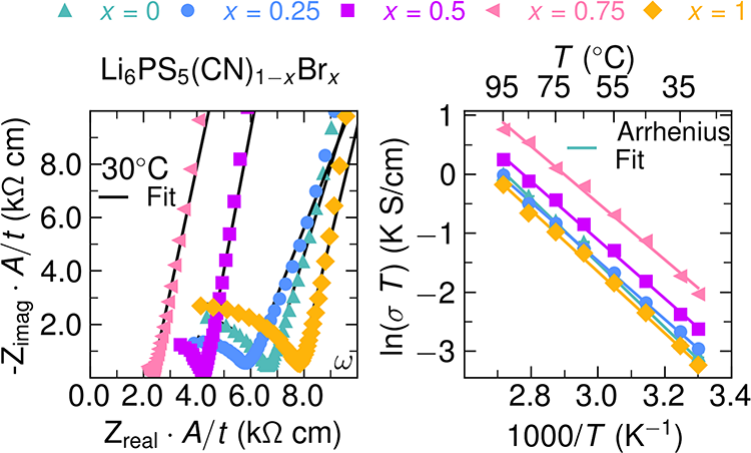
(a) Representative Nyquist plots of Li_6_PS_5_(CN)_1–*x*_Br_*x*_ at *T* = 30 °C. The real and imaginary impedance axes are normalized by area (*A*) and thickness (*t*) of pellets. Data points are represented by markers, and the solid lines indicate the (*R*_1_ *Q*_1_) + *Q*_2_ equivalent circuit fits. (b) Representative Arrhenius relationships across the Li_6_PS_5_(CN)_1–*x*_Br_*x*_ series determined from temperature-dependent electrochemical impedance spectroscopy. Linear regressions are shown as solid lines. Error bars represent the standard deviation between three replicate frequency sweeps at each temperature, and are generally smaller than the markers. Arrhenius plots for all sample replicates are included in Figure S20.

We observe a nonmonotonic change in room-temperature Li^+^ conductivity across the series, as shown in Figure 5a. The Li_6_PS_5_CN and Li_6_PS_5_Br end members exhibit room temperature ionic conductivities of σ_30°C_ = 1.56(4) × 10^–4^ S cm^–1^ and σ_30°C_ = 1.289(1) × 10^–4^ S cm^–1^, respectively. The bulk ionic conductivity of the low-temperature solution-phase synthesized Li_6_PS_5_Br is lower than prior reports using traditional solid-state synthesis.^46^ We attribute this difference to greater contributions from grain-boundary resistance due to smaller particle sizes in the low-temperature method, as observed previously.^27,41^ The measured lithium ion conductivity for Li_6_PS_5_CN is higher than our previous studies (6(2) × 10^–5^ S cm^–1^ and 6.8(2) × 10^–5^ S cm^–1^),^27,28^ which may be attributed to differences between synthesis batches or electrochemical cell setup; variability is common in measured properties of solid-state electrolytes, particularly for materials prepared by low-temperature solution-phase routes.^41,47,52^ For *x* = 0.25, we observe similar ionic conductivity at 30 °C to the two end members. However, at *x* = 0.5 and 0.75, the lithium-ion conductivity increases and reaches a maximum at σ_30°C_ = 4.0(4) × 10^–4^ S cm^–1^ for *x* = 0.75. We note that the differences in ionic conductivity—while statistically significant —are fairly small across the series.

**Figure 5:**
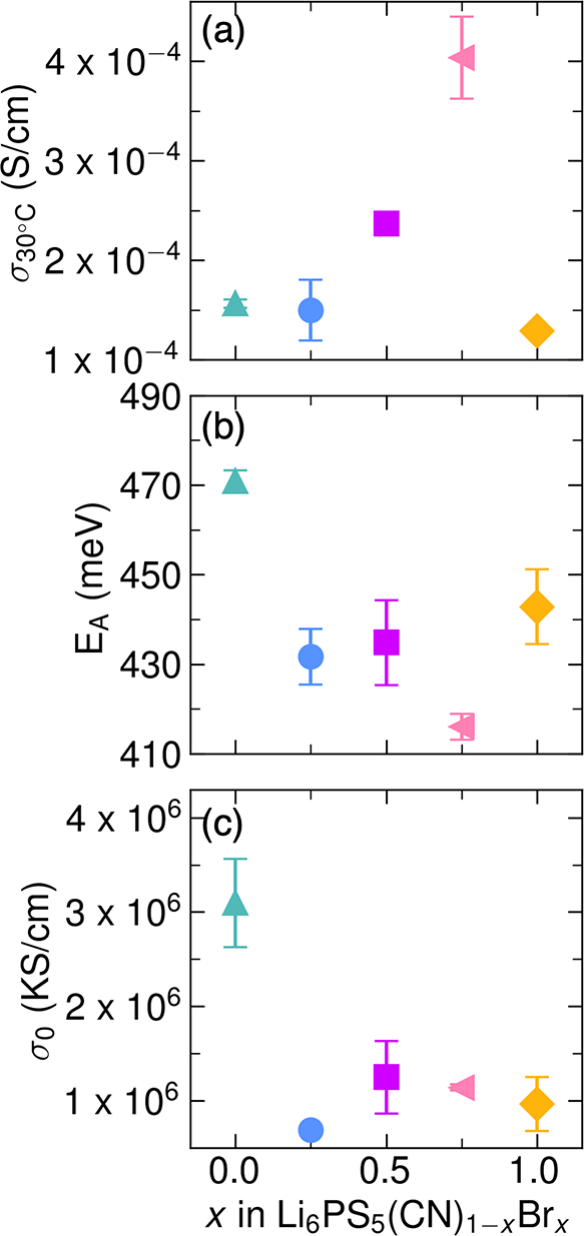
(a) Ionic conductivity at 30 °C (σ_30°C_), (b) activation energy (*E*_A_), and (c) the temperature-independent Arrhenius prefactor (σ_0_) as a function of *x* in Li_6_PS_5_(CN)_1–*x*_Br_*x*_. Error bars show the standard deviation between measurements for two replicate pellets. Data are also shown in Table S4.

Temperature-dependent EIS measurements provide further insight into the underlying contributions to bulk Li^+^ transport in the Li_6_PS_5_(CN)_1–*x*_Br_*x*_ series. Temperature-dependent EIS data and Nyquist plots for all compositions are included in the Supporting Information (Tables S5–S14 and Figures S10–S19). As shown in Figure 4b, Li^+^ conductivity for all members of the series follows the Arrhenius relationship σ*T* = σ_0_exp­(−*E*_A_/*k*_B_ *T*) over all measured temperatures. From Arrhenius fits to the temperature-dependent conductivity, we extracted the activation energy (*E*_A_) and the temperature-independent prefactor to the conductivity (σ_0_).

Substitution of Br^–^ with CN^–^ results in a decrease in the activation barrier across the solid solution. For example, 25% Br^–^ substitution reduces the activation barrier from 471(2) meV for Li_6_PS_5_CN to 432(6) meV. The activation barrier is further reduced to 416(3) meV for *x* = 0.75, which corresponds to the composition with the highest Li^+^ conductivity (Figure 5a,b). For the Li_6_PS_5_Br end member, we observe a slightly higher activation barrier than members of the series, which suggests that extensive anion substitution moderates the energetic landscape for ion hopping. Further, we find that the Arrhenius prefactor is highest for Li_6_PS_5_CN (3.1(5) × 10^6^ KS cm^–1^) and decreases substantially to 6.9(4) × 10^5^ KS cm^–1^ for *x* = 0.25 (Figure 5c). Increasing substitution of bromide across the series does not appear to strongly impact the Arrhenius prefactor; this observation will be discussed in greater detail in a subsequent section.

### Impact of Anion Site Disorder on Ion Transport

Site mixing between S^2–^ and (pseudo)­halides on the 4*a* and 4*d* Wyckoff positions has long been correlated with fast ion transport in the argyrodites.^1,6,10,12–16,28,46,53^ While quantifying anion site disorder is straightforward for many argyrodite compositions, the Li_6_PS_5_(CN)_1–*x*_Br_*x*_ series presents a unique situation where three anions contribute to the overall site mixing. As discussed in the Structure section (*vide supra*), the combination of anion site mixing and the asymmetry of the cyanide dipole contributes additional complexity to the anion local structure. In order to quantify how the degree of S^2–^/CN^–^/Br^–^ site mixing impacts lithium ion transport in the Li_6_PS_5_(CN)_1–*x*_Br_*x*_ series, we calculated the configurational entropy (*S*_config_) based upon the refined anion occupancies from the SXRD data. *S*_config_ has previously been employed to quantify site disorder in mixed-halide argyrodites,^19,21^ and can be approximated for the *4a* and *4d* Wyckoff sites via

1 $$\frac{S_{\mathrm{config}}}{R}\approx -\sum_{i}x_{i}\ln x_{i}$$

where *R* is the ideal gas constant, *i* represents each of the three anions on the *4a* and *4d* sites, and *x*_*i*_ is the refined occupancy for that species on that site.^54^ While we expect that orientational disorder of cyanide dipoles would result in additional configurational entropy for *x* < 1, the lack of information about preferred orientations of cyanide precludes finite enumeration of orientational states.

The ionic conductivity across the Li_6_PS_5_(CN)_1–*x*_Br_*x*_ series at *T* = 30 °C appears to be correlated with *S*_config_, as shown in Figure 6a. The *4a* and *4d* sites exhibit maximum anion mixing of S^2–^, CN^–^, and Br^–^ at *x* = 0.75 (Figure 6b), which leads to the highest *S*_config_ and highest ionic conductivity. To unravel the relationship between bulk ionic conductivity and triple-anion site mixing, we investigated contributions to ionic conductivity in the context of *S*_config_. We find that the activation energy is also correlated with the configurational entropy; *E*_A_ is minimized as σ_30°C_ reaches a maximum for *x* = 0.75 (Figure S21). This behavior is not unique to the dipole-diluted argyrodite series, as configurational entropy is correlated with a reduction in *E*_A_ that drives an increase in Li^+^ transport in a variety of halide argyrodite substitution series.^18,19,21^

**Figure 6:**
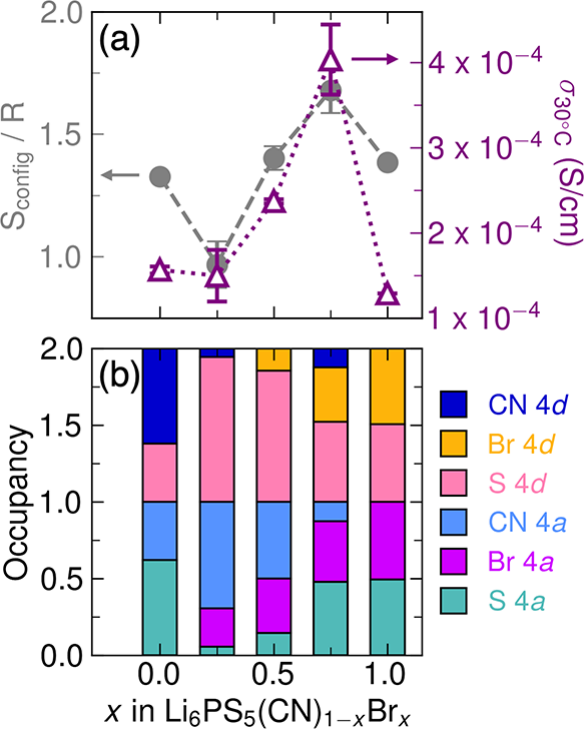
(a) Configurational entropy (*S*_config_) normalized by the ideal gas constant (gray, left axis) and ionic conductivity at *T* = 30 °C (purple, right axis) follow similar trends across *x* in Li_6_PS_5_(CN)_1–*x*_Br_*x*_. Error bars for *S*_config_ represent error for anion occupancies on *4a* and *4d* propagated through eq 1. Error bars for σ_30°C_ show the standard deviation between electrochemical impedance spectroscopy measurements of ionic conductivity between two pellets. (b) The *x* = 0.75 composition exhibits anion occupancies on 4*a* and 4*d* closest to maximum spatial distribution of CN^–^, Br^–^, and S^2–^.

As the activation energy and ionic conductivity are strongly correlated with the degree of anion site mixing quantified by *S*_config_, we hypothesize that the distribution of anions that maximizes *S*_config_ also diversifies the local coordination environment for Li^+^ ions. In turn, we propose that the local variability of the Coulombic interactions lowers the activation barriers for bulk ion transport. The onset of anion site disorder in halide argyrodites with a single halide anion results in a large reduction in activation energy.^6,46^ Prior molecular dynamics simulations and neutron diffraction studies indicate that the onset of anion site disorder opens pathways for intercage lithium ion hopping through locally randomized spatial distribution of anion charge, which may contribute to this reduction in E_A_.^14,15^ In Li_6_PS_5_(CN)_1–*x*_Br_*x*_, the nonspherical charge density of CN^–^ adds an additional layer of complexity to the electrostatic interactions. While Br^–^ and CN^–^ have similar charges and ionic radii, the volumetric charge distribution between the two anions is very different: Br^–^ has a spherical charge distribution, whereas CN^–^ has a dumbbell-shaped distribution with the dipole moment μ pointing toward the carbon (Figure 1a). Additionally, our previous molecular dynamics simulations of Li_6_PS_5_CN indicate that Li^+^ preferentially resides near the carbon end of the CN^–^ dipole, highlighting the impact of spatial charge distribution in this materials family.^28^ While we cannot definitively resolve the relative positions of Li^+^ and CN^–^ from the structural studies presented here, the scattering data are consistent with orientationally disordered cyanide anions. Even in the case of ordered cyanide orientations, the asymmetric charge distribution of the cyanide dipole introduces a new dimension of disorder to the argyrodite structure. Therefore, we propose that site mixing between three anions with distinct charges and form factors drives local variability of the energetic landscape to lower the activation energy for ion transport.

### Decoupling of Arrhenius Prefactor and Activation Energy

In many fast ion conductors, reducing the activation energy is accompanied by an exponential reduction in the Arrhenius prefactor. This phenomenon is commonly referred to as the Meyer-Neldel^55^ rule and originates from entropy-enthalpy compensation; in an activated process, a larger enthalpic barrier requires assembling a larger number of excitations to overcome the barrier (e.g., higher entropy).^56–59^ In the Meyer-Neldel regime, compensatory behavior imposes an upper bound on macroscopic ionic conductivity. Breaking the Meyer-Neldel rule therefore offers a potential pathway to surpass this limit. As shown in Figure 7a, several families of multianion argyrodites follow a fairly linear relationship of ln­(σ_0_) vs *E*_A_ according to the Meyer-Neldel rule.^6,17–19^ However, the prefactor and activation energy appear to be decoupled in Li_6_PS_5_(CN)_1–*x*_Br_*x*_ (Figure 7b), suggesting that cyanide may play a role in the deviation from Meyer-Neldel behavior.

**Figure 7:**
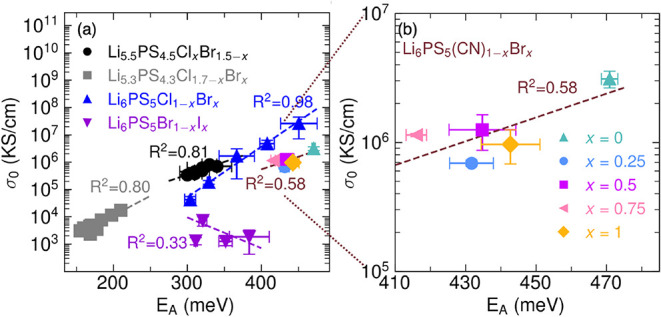
(a) Relationship between Arrhenius prefactor (σ_0_) and activation energy (*E*_A_) for mixed-halide argyrodites and Li_6_PS_5_(CN)_1–*x*_Br_*x*_. Data reproduced from refs 6,18,19 . (b) Zoom region for the Li_6_PS_5_(CN)_1–*x*_Br_*x*_ data. Error bars for *E*_A_ and σ_0_ represent the standard deviations between two replicate EIS measurements.

In order to better understand the impact of the cyanide anions on the apparent decoupling of the prefactor and activation energy, we used heat capacity measurements to quantify the attempt frequency term (ν_0_) within the prefactor. The prefactor is given by

2 $$\sigma_{0}=\frac{\gamma c\nu_{0}Z^{2}e^{2}a_{0}^{2}\exp\left(\Delta S_{m}\right)}{k_{B}}$$

where γ is a geometric factor that is taken to be 1/3 for cubic symmetry,^60^ *c* is the carrier concentration, *Z* is the charge of the ion (1), e is the elementary charge constant, *a*_0_ is the jump distance, Δ*S*_m_ is the entropy of migration, and *k*_B_ is the Boltzmann constant.^61^ As the attempt frequency (ν_0_) approximated via the Rice-Roth equation^60^ has previously been shown to correlate with the Debye frequency (ν_D_) obtained from speed of sound measurements,^6^ ν_D_ is often taken as an approximation for ν_0_.^62–64^ The Debye model describes heat capacity in the low temperature limit through

3 $$C_{P}\approx \frac{12Nk_{B}\pi^{4}}{5}{\left(\frac{T}{\Theta_{D}}\right)}^{3}$$

where *N* is Avogadro’s number times the number of atoms per formula unit, *T* is temperature, and Θ_D_ is the Debye temperature.^65^ Moreover, Θ_D_ is directly related to ν_D_ via $\nu_{D}=\frac{\Theta_{D}k_{B}}{h}$ , where *h* is Planck’s constant.^66^ Through Debye model fits to low-temperature heat capacity measurements, we extracted ν_D_ for Li_6_PS_5_(CN)_1–*x*_Br_*x*_ (Figure 8). The Debye frequency represents the maximum frequency of the acoustic phonon modes at the Brillouin zone edge in a linear dispersion model, and is used as a proxy for lattice softness.^67^ ν_*D*_ is on the order of 1 × 10^12^ Hz for all compositions, which is consistent with previous reports of ν_D_ and ν_0_ in argyrodites.^6,17,68^ While lattice softness has been extensively investigated as a major driving force for improved ionic transport,^6,63,64,69^ Figure S23 shows that ν_D_ does not seem to correlate with σ_30°C_, *E*_A_, or σ_0_.

**Figure 8:**
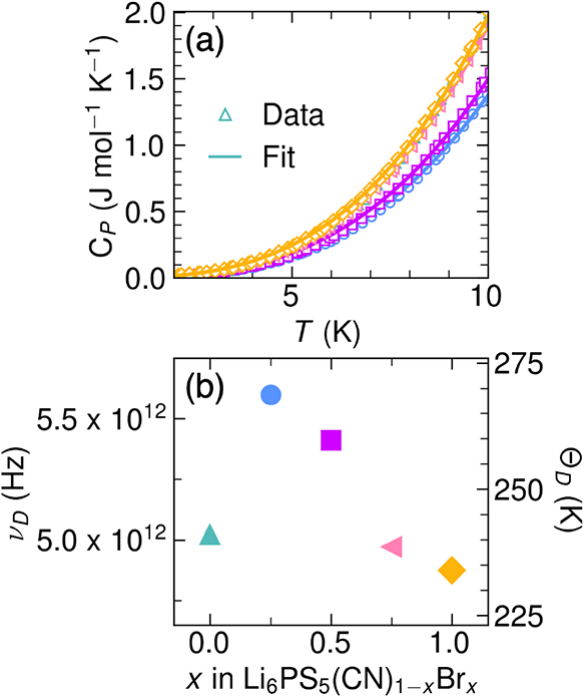
(a) Molar heat capacity at constant pressure (*C*_P_, markers) with the Debye fit (solid lines, eq 3) for 2 to 10 K. *C*_P_ from 2 to 250 K is shown in Figure S22. (b) Debye frequency (ν_D_, Hz, left axis) and Debye temperature (Θ_D_, *K*, right axis) extracted from the Debye fit to low temperature heat capacity (eq 3) as a function of *x* in Li_6_PS_5_(CN)_1–*x*_Br_*x*_.

Since the Debye frequency does not fully explain the behavior of the prefactor in the dipole-diluted series, we find that the entropy of migration is the most influential of the remaining factors in eq 2. The jump distance (*a*_0_) in argyrodites is typically determined from neutron diffraction data as the distance between the Li^+^ cages surrounding the 4*d* sites.^17^ While we lack neutron diffraction data suitable for Rietveld refinement of the Li^+^ sublattice in Li_6_PS_5_(CN)_1–*x*_Br_*x*_, previous reports indicate that *a*_0_ varies between ∼1.8 and ∼2.3 Å in Li_6–*x*_PS_5–*x*_Br_1+*x*_.^17^ It is possible that the variability of the anion spatial charge distribution in Li_6_PS_5_(CN)_1–*x*_Br_*x*_ would induce a spatial redistribution of Li^+^, but *a*_0_ would likely be limited to a similarly small range. Likewise, previous reports suggest that the carrier concentration *c* is on the order of magnitude of 1 × 10^22^ cm^–3^ in Li_6_PS_5_X argyrodites.^68^ Aliovalent doping is a known handle to increase *c* in solid electrolytes through the generation of interstitial sites or vacancies.^70,71^ However, the isovalent substitution between CN^–^ and Br^–^ in this study is unlikely to change the carrier concentration significantly, beyond small changes to the enthalpy of defect formation in the intrinsic regime. Finally, the last variable in eq 2 is the entropy of migration (Δ*S*_m_). Defined as the difference in entropy between the transition state and the initial state for a mobile cation, Δ*S*_m_ also depends on the ratio of the product over the vibrational frequencies of *N* – 1 normal modes of the initial state (ν^0^) and the transition state (ν′)^72,73^

4 $$\Delta S_{m}=k_{B}\ln\left(\frac{\overset{N-1}{\underset{j=1}{\Pi }}\nu_{j}^{0}}{\overset{N-1}{\underset{j=1}{\Pi }}\nu_{j}^{\prime }}\right)$$

We calculated Δ*S*_m_ from σ_0_ according to eq 2, as shown in Figure 9. We approximated the attempt frequency as ν_D_ from the low temperature Debye fits to the heat capacity (Figure 8b). Across physically reasonable ranges of *a*_0_ and *c* values, Δ*S*_m_ is clearly maximized for *x* = 0.

**Figure 9:**
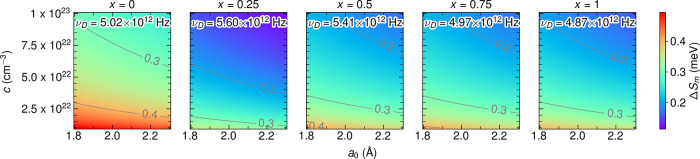
Δ*S*_m_ (meV) calculated using eq 2 from σ_0_ extracted from Arrhenius fits to temperature dependent EIS measurements (Figure 4b) and ν_0_ ∼ ν_D_ extracted from Debye fits to low temperature heat capacity measurements (Figure 8), as a function of possible carrier concentrations (*c*) and jump distances (*a*_0_) across *x* in Li_6_PS_5_(CN)_1–*x*_Br_*x*_.

We propose that stronger Li-dipole Coulombic interactions caused by the higher volumetric charge density of cyanide increases the vibrational frequencies in the initial state, raising the entropy of migration for *x* = 0. There is a significantly larger quantity of CN^–^ on *4d* in Li_6_PS_5_CN (0.62 equiv *4d* occupancy) relative to all other compositions (Table 1). The Li^+^ cages in the argyrodite structure are generally considered as the initial, stable sites for the hopping pathway.^14^ As the *4d* site sits at the center of the Li^+^ cages (Figure 1a), Li^+^-CN^–^ electrostatic interactions will have a more significant impact on the stable site vibrational frequencies (ν_*j*_^0^ in eq 4) in the *x* = 0 composition. Though the ionic radii of CN^–^ and Br^–^ are similar (1.87 and 1.96 Å, respectively),^44,45^ the highly directional bonding within the CN^–^ molecule results in a significant reduction in anion volume in comparison to Br^–^. Geometry optimizations using the Møller–Plesset 2 level of theory show that the volumetric charge density (ρ) of CN^–^ is 0.424 e/Å^3^, whereas ρ = 0.0317 e/Å^3^ for Br^–^.^44,74^ This order of magnitude increase in the charge density drives stronger CN^–^-Li^+^ Coulombic interactions. As mentioned previously, our prior molecular dynamics study shows that Li^+^ preferentially occupies sites near both ends of the cyanide dipole, providing additional evidence for strong electrostatic coupling between CN^–^ and Li^+^.^28^ We argue that stiffer interactions between CN^–^ on 4*d* and the surrounding Li^+^ cage increases stable-site Li^+^ vibrational frequencies, driving an increase in the entropy of migration for *x* = 0. This trend can be observed in the relationship between the Arrhenius prefactor normalized by the Debye frequency and CN^–^ occupancy on *4d* in Figure S24. While we recognize that the limited range of data points with significant CN^–^ occupancy on the 4*d* site prevents conclusive correlations, the observed trend supports our theoretical considerations of the impact of dipolar-cation interactions on vibrational frequencies and entropy of migration.

Across a range of materials, “breaking Meyer-Neldel” can be explained by electrostatic and geometric tuning of the vibrational dynamics of the initial and transition Li^+^ states. A previous *ab initio* molecular dynamics study of LiTi_2_(PS_4_)_3_ found that changes to the vibrational frequencies of the stable and activated Li^+^ states also increased the entropy of migration and decoupled the Arrhenius prefactor from the activation energy.^75^ Likewise, calculations of the phonon density of states using density functional theory show that geometric constraints of the initial Li^+^ sites increase the vibrational energies and Δ*S*_m_ without raising *E*_A_ in Li_3_OCl_1–*x*_Br_*x*_.^69^ In contrast, halide argyrodites with consistent levels of *4a*/*4d* site-disorder usually exhibit Meyer-Neldel behavior, as can be seen in Figure 7a (black circles, gray squares, and blue triangles).^6,18,19^ However, σ_0_ and *E*_A_ are decoupled in the substitution series Li_6_PS_5_Br_1–*x*_I_*x*_ (purple triangles), which has historically been credited to the onset of site-disorder upon addition of Br^–^.^6^ In Li_6_PS_5_I, S^2–^ occupies the 4d site almost exclusively (0.966(4)-1),^6,46^ resulting in stronger Coulombic interactions between the divalent anion and the surrounding Li^+^ cage.^15^ We propose that strong Coulombic interactions between high charge density anions and stable Li^+^ sites result in increased ν_*j*_^0^, Δ*S*_m_, and σ_0_ for the full iodide argyrodite, according to eqs 2 and 4, consistent with our findings in Li_6_PS_5_(CN)_1–*x*_Br_*x*_. Clearly, the vibrational properties of the Li^+^ initial and transition states emerge as an opportunity for decoupling σ_0_ and *E*_A_ to achieve fast ion transport in the solid state.

### Conclusion

We prepared the substitution series Li_6_PS_5_(CN)_1–*x*_Br_*x*_ via a low-temperature, solution-phase synthesis. Cubic argyrodite *F*4̅3*m* symmetry and solid-solution formation was confirmed by synchrotron powder X-ray diffraction and neutron powder diffraction studies. In-depth structural analysis is consistent with complex anion site mixing coupled with orientational disorder of the cyanide anions. We find that extensive triple-anion site mixing in Li_6_PS_5_(CN)_0.25_Br_0.75_ minimizes the activation barrier for lithium migration and maximizes room temperature ionic conductivity. We propose that differences in the spatial charge density due to anion site mixing and cyanide asymmetry moderate the energetic landscape for ion hopping in the Li_6_PS_5_(CN)_1–*x*_Br_*x*_ series. Furthermore, we find that substitution of cyanide with bromide in this series decouples the changes in the activation barrier from the Arrhenius prefactor. We rationalize this “breaking” of the Meyer-Neldel rule by considering how the dipolar cyanide anions affect vibrational properties of the surrounding structure and the entropy of migration. This work presents configurational complexity—including site mixing and potential for orientational disorder—as a key design principle for tuning both the enthalpic and entropic contributions to ion transport in pseudohalide argyrodites.

## Supplementary Material



## Notes

Crystal structures, synthesis details, additional synchrotron X-ray powder diffraction refinement details, internal standard synchrotron X-ray powder diffraction data, *R*_wp_ heat maps, neutron powder diffraction data and refinement details, Raman and Fourier-transform infrared spectra and analysis, Nyquist plots, Arrhenius plots, AC electrochemical impedance spectroscopy equivalent circuit fit parameters, *S*_config_ vs *E*_A_, heat capacity data, ν_D_ vs σ_30*°*C_, *E*_A_, and σ_0_, and CN^–^ occupancy on *4d* vs σ_0_/ν_D_. Raw data and Python analysis codes are available in the Maughan-Lab/Expanding-Configurational-Complexity GitHub repository (PDF)
