# Supplementary material for: Expanding Configurational Complexity through Dipole Dilution in Pseudohalide Argyrodite Ion Conductors
Source: Chem Mater. 2025 Dec 4;37(24):9787–98. doi: 10.1021/acs.chemmater.5c02236 (PMC12746411; doi:10.1021/acs.chemmater.5c02236)
Supplement: Supplementary file 1 [file cm5c02236_si_001.pdf]

Supporting Information for "Expanding Configurational Complexity through Dipole Dilution in Pseudohalide Argyrodite Ion Conductors"

Shelby L. Galinat,† Claire Willard,‡ Krishna Teja Valeti,¶ Rebecca W. Smaha,§ Anna Staerz,¶ and Annalise E. Maughan*,‡

†*Materials Science Program, Colorado School of Mines, Golden, Colorado 80401, United States*

‡*Department of Chemistry, Colorado School of Mines, Golden, Colorado 80401, United States*

¶*Department of Metallurgical and Materials Engineering, Colorado School of Mines, Golden, Colorado 80401, United States*

§*National Renewable Energy Laboratory, Golden, CO 80401, United States*

E-mail: amaughan@mines.edu

Structure of $Li_6PS_5(CN)_{1-x}Br_x$

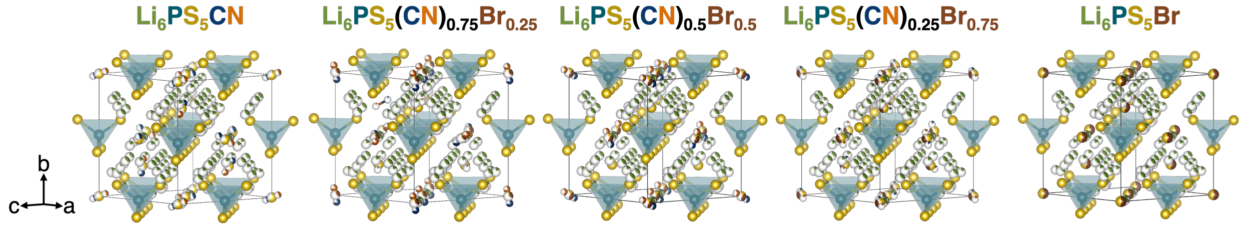

Figure S1: Pseudocubic ($P1$) structural models of $Li_6PS_5(CN)_{1-x}Br_x$ displaying random orientational disorder of CN^- dipoles for $x \leq 1$.

Solution Phase Synthesis of $Li_6PS_5(CN)_{1-x}Br_x$

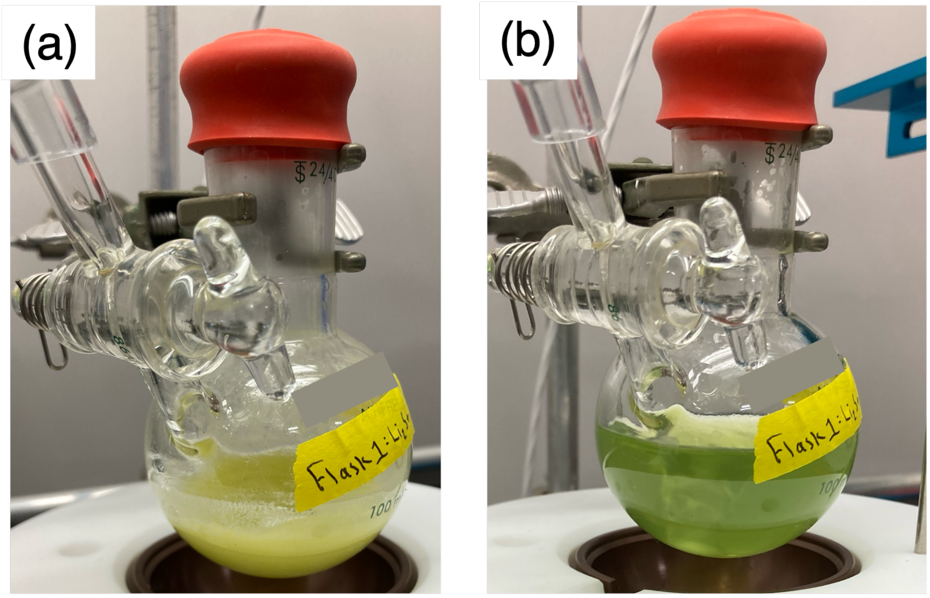

Figure S2: (a) After stirring for 1.25 hours, the $1P_2S_5$:$3Li_2S$ suspension in tetrahydrofuran (THF) turns a sunny yellow color. (b) The solution immediately turns a transparent green color upon addition of the ethanol (EtOH) solution with $2Li_2S$:$2LiX$ (X = CN, Br).

Rietveld Refinements of Synchrotron Powder X-ray Diffraction

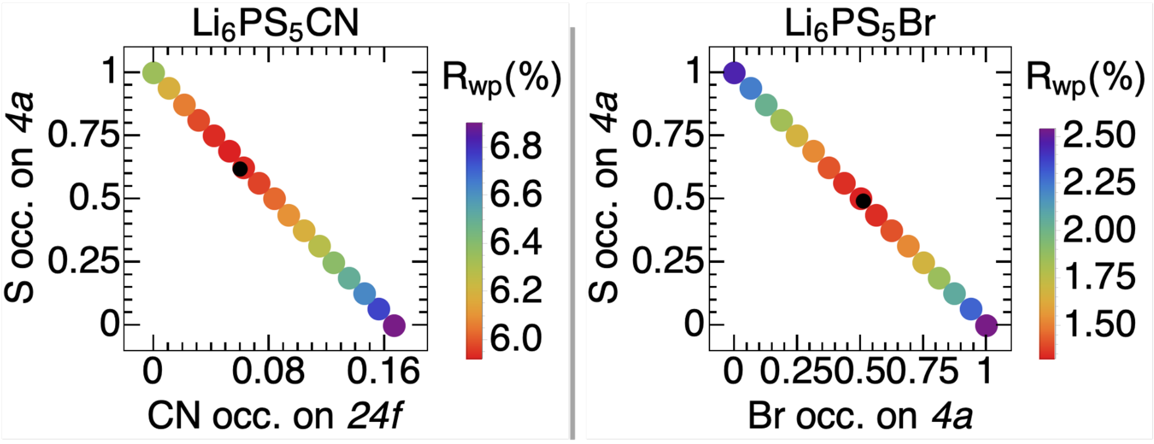

Figure S3: R_{wp} heat maps for S^{2-}/X^- (X = CN^-, Br^-) fixed occupancies on the 24*f* (CN^-) and 4*a* (S^{2-}, Br^-) sites for Li_6PS_5CN and Li_6PS_5Br. The black circles represent freely refined values.

Table S1: Zero shift (λ = 0.73 Å), volume-weighted mean column length particle size metric from integral-breadth peak broadening fitting (L_{vol}), ε_0 strain, fractional coordinate position of S on 16*e*, and the isotropic displacement parameters for the S on 16*e* and P from Rietveld refinement of synchrotron X-ray diffraction of powder samples of $Li_6PS_5(CN)_{1-x}Br_x$ (x = 0, 0.25, 0.5, 0.75, 1). After refining the lattice parameters against the fixed lattice parameter of the silicon internal standard for the samples including internal standards (see Fig. S5), the zero shift was refined against the fixed lattice parameter for the samples without internal standards.

x	zero shift (°2θ)	L_{vol} (nm)	ε_0	S 16*e* pos.	S 16*e* U_{iso} (Å^2)	P U_{iso} (Å^2)
0	0.0054(2)	32.2(6)	0.00038(2)	0.1248(2)	0.0611(8)	0.067(2)
0.25	0.0022(1)	36.7(5)	0.00044(2)	0.1237(1)	0.0646(6)	0.055(1)
0.5	0.0022(1)	35.7(5)	0.00066(2)	0.1219(1)	0.0595(6)	0.062(1)
0.75	0.0014(1)	32.8(5)	0.00089(2)	0.1205(1)	0.0584(8)	0.076(2)
1	0.0004(2)	41.4(9)	0.00124(2)	0.1189(2)	0.0528(9)	0.089(2)

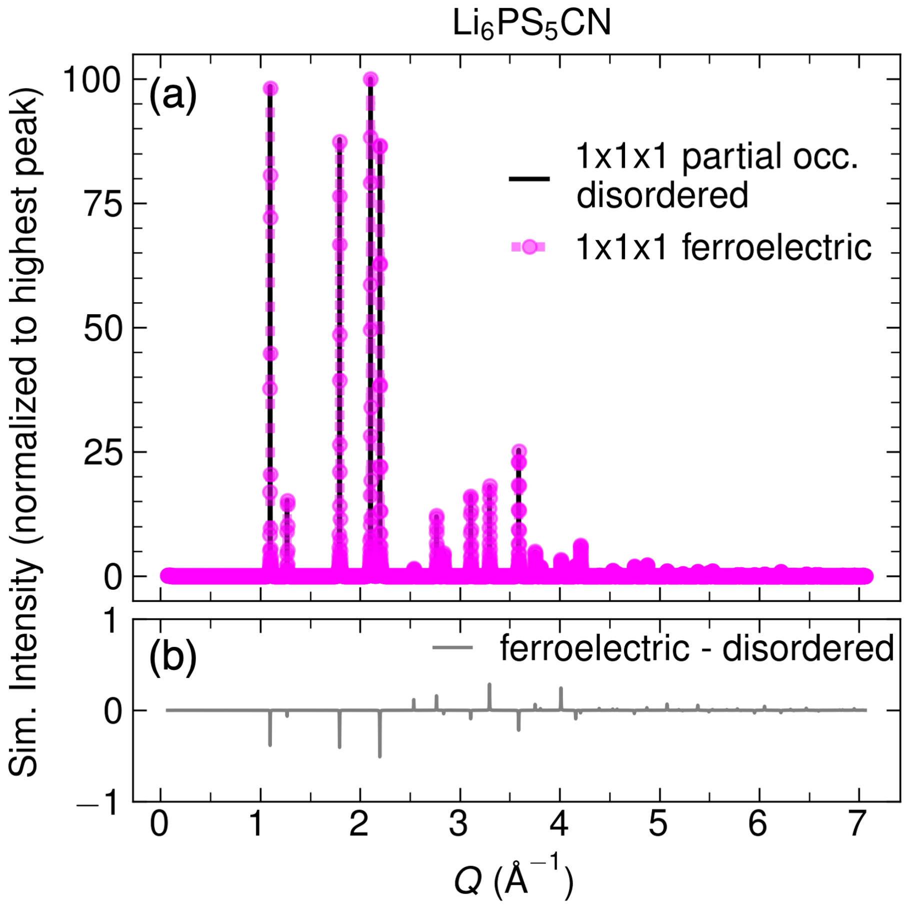

Figure S4: (a) Simulated X-ray diffraction patterns in VESTA using single unit cell models with different CN^- orientational order in Li_6PS_5CN. The partial occupancy, disordered, $F\bar{4}3m$ and the $P1$ ferroelectrically-ordered CN^- simulated patterns are shown with a black line and pink dotted line, respectively. (b) Subtracting the disordered from the ferroelectric simulated pattern shows that the differences in the intensity due to CN^- ordering are ~ 1 % of the overall intensity.

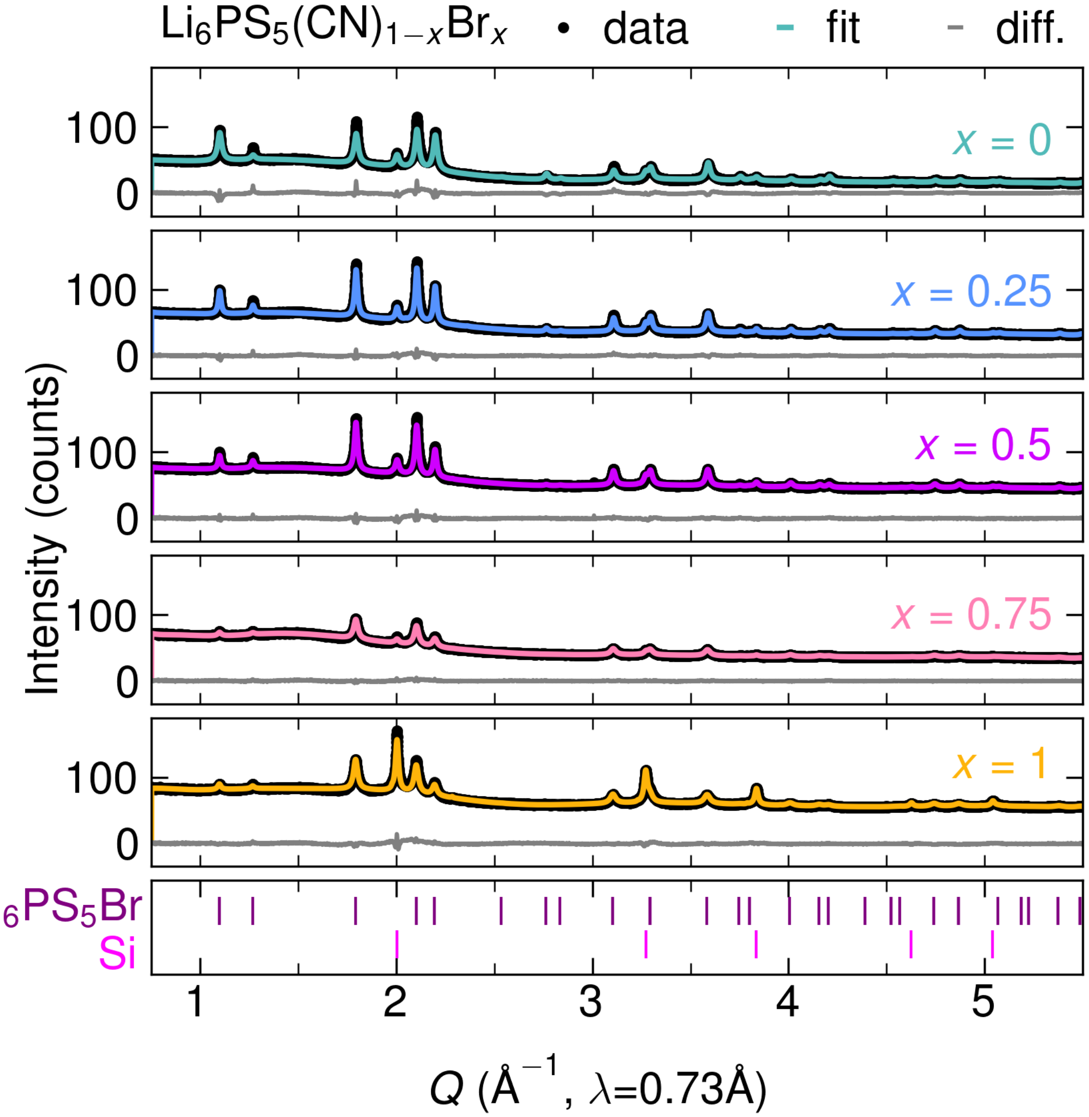

Figure S5: Rietveld refinements of the cubic argyrodite structure (space group $F\bar{4}3m$) and a Si internal standard with room temperature synchrotron powder X-ray diffraction (SXRD) data for $Li_6PS_5(CN)_{1-x}Br_x$ (x = 0, 0.25, 0.5, 0.75, 1) collected at the Stanford Synchrotron Radiation Lightsource (SSRL) at SLAC National Accelerator Laboratory on beamline BL2-1 with wavelength λ = 0.73 Å. Data are shown as black circles, fits are shown as colored lines, and difference curves are shown as gray lines. The purple and pink tick marks show the positions of expected reflections for the cubic argyrodite structure and the Si internal standard, respectively. ∼10 wt% Si was added to samples for $0 \leq x \leq 0.75$ and ∼35 wt% Si was added to the $x = 1$ sample.

Neutron Powder Diffraction

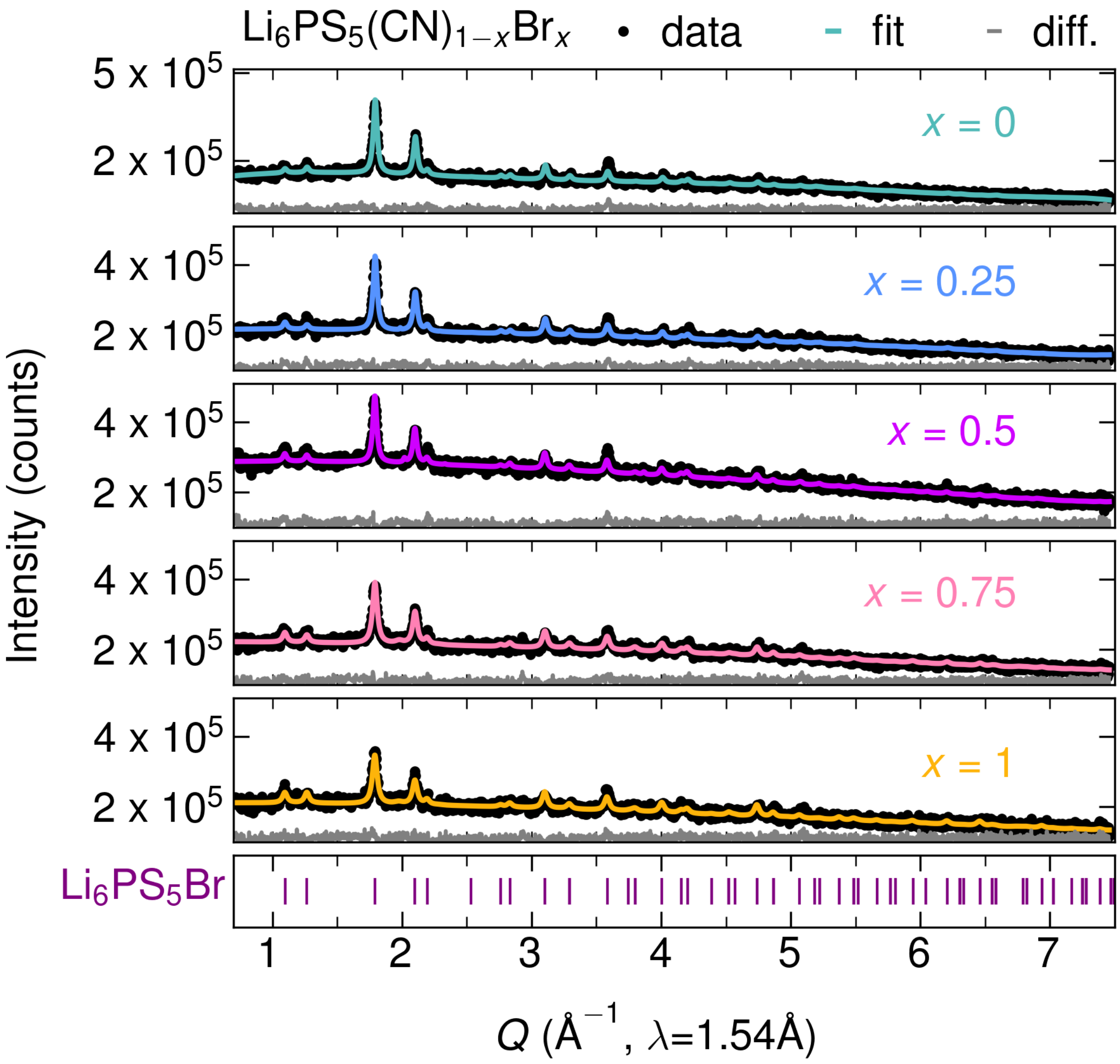

Figure S6: Rietveld refinements of the cubic argyrodite structure (space group $F\bar{4}3m$) with room temperature neutron powder diffraction (NPD) data for $Li_6PS_5(CN)_{1-x}Br_x$ (x = 0, 0.25, 0.5, 0.75, 1) collected at the High Flux Isotope Reactor (HFIR) at Oak Ridge National Laboratory (ORNL) on beamline HB-2A with wavelength λ = 1.54 Å. Data are shown as black circles, fits are shown as colored lines, and difference curves are shown as gray lines. The purple tick marks show the positions of expected reflections for the cubic argyrodite structure.

Table S2: Occupancies for S, CN, and Br from Rietveld refinement of neutron diffraction of powder samples of $Li_6PS_5(CN)_{1-x}Br_x$ (x = 0, 0.25, 0.5, 0.75, 1).

x	S 4a	CN 24f	Br 4a	S 4d	CN 16e	Br 4d
0	0.54(2)	0.076(3)	NA	0.4587(2)	0.135(4)	NA
0.25	0.54(6)	0.0427(3)	0.21(8)	0.465(3)	0.1234(6)	0.042(7)
0.5	0.40(5)	0.0160(1)	0.50(8(	0.596(3)	0.1010(2)	0.00(8)
0.75	0.46(5)	0.0026(2)	0.53(7)	0.543(2)	0.0586(4)	0.222(5)
1	0.52(4)	NA	0.482(1)	0.482(1)	NA	0.518(1)

Table S3: Lattice parameter, volume-weighted mean column length particle size metric from integral-breadth peak broadening fitting (L_{vol}), Li occupancies, and R weighted pattern (R_{wp}) values from Rietveld refinement of neutron diffraction of powder samples of $Li_6PS_5(CN)_{1-x}Br_x$ (x = 0, 0.25, 0.5, 0.75, 1).

x	a (Å)	L_{vol} (nm)	Li T5 (48h) occ.	Li T5a (24g) occ.	Li T2 (48h) occ.	R_{wp} (%)
0	9.904(1)	11.6(3)	0.50(3)	0.00(5)	0(10)	3.3353
0.25	9.909(1)	11.1(3)	0.50(2)	0.00(5)	0(20)	3.5506
0.5	9.922(2)	11.6(4)	0.50(3)	0.00(5)	0(20)	3.3186
0.75	9.918(2	9.7(3)	0.50(2)	0.00(4)	0(20)	3.7418
1	9.923(2)	9.4(4)	0.50(2)	0.00(5)	0(20)	4.4582

Infrared and Raman Spectroscopy

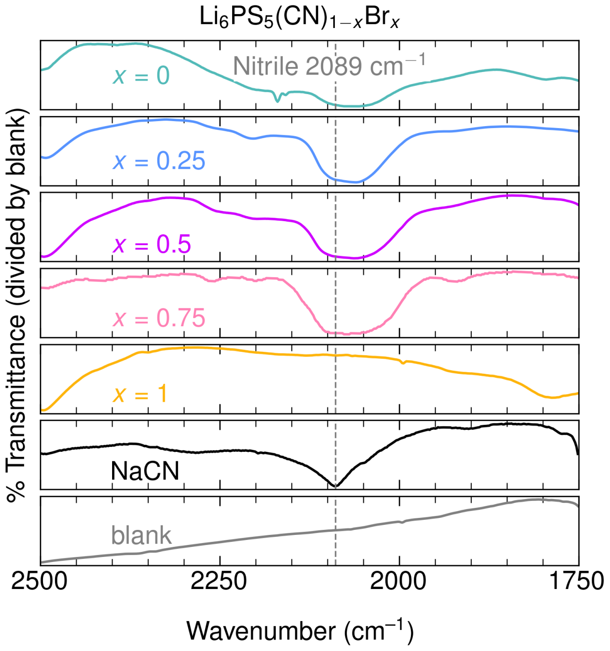

Figure S7: Transmission Fourier-transform infrared (FT-IR) spectra for $Li_6PS_5(CN)_{1-x}Br_x$, NaCN, and a KBr blank. The location of the CN^- nitrile stretch at ~2089 cm^{-1} is denoted by the dashed gray line.

Transmission Fourier-transform infrared (FT-IR) spectroscopy reveals vibrational modes consistent with the cyanide anion in all cyanide-containing compounds. The FT-IR spectra shown in Figure S7 reveal a broad nitrile stretch at 2089 cm^{-1} for NaCN and all compositions where $x < 1$. In contrast, the spectra of Li_6PS_5Br and the KBr blank do not exhibit this feature, which lends support to our assertion that cyanide is successfully incorporated across the substitution series. In $Li_6PS_5(CN)_{1-x}Br_x$, the nitrile stretch is asymmetrically broadened towards lower wavenumbers with an average full width at half minimum (FWHM) of ~115 cm^{-1}. This broadening may indicate a diversity of local CN^- environments produced by site disorder, dipole dilution, and/or orientational disorder. For example, dilute CN^- anions in KBr undergo dynamic reorientations at room temperature and exhibit a broad nitrile stretch with a FWHM of ~ 50 cm^{-1}.[1,2] Similarly, in CN^- containing perovskites, disordered CN^- orientations are correlated with broadened nitrile stretches.[3] Alternatively, broadening of IR stretches is known to be influenced by the geometry of

the measurement.[4] However, the nitrile stretch for the NaCN positive control is much narrower, which lends support to the hypothesis that the cyanide anions exhibit a distribution of distinct environments in the $Li_6PS_5(CN)_{1-x}Br_x$ series.

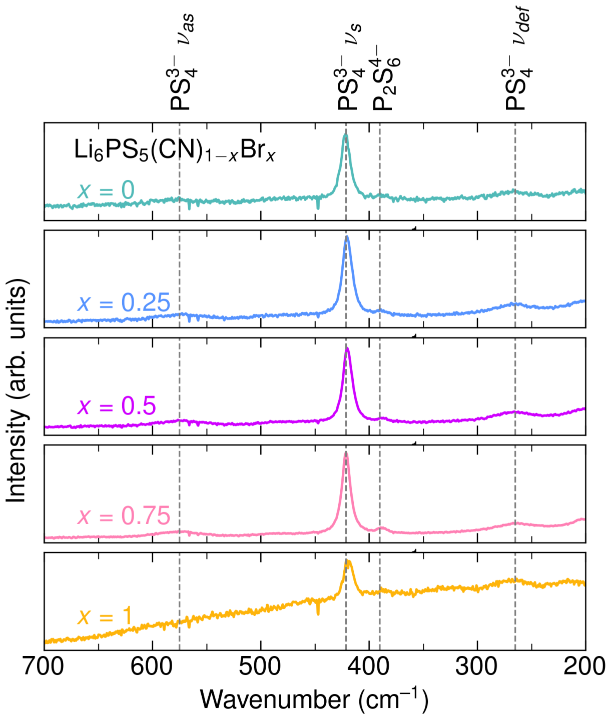

Figure S8: Baseline-subtracted Raman spectra for $Li_6PS_5(CN)_{1-x}Br_x$. The assigned vibrational modes are labeled with dashed grey lines.

We identify thiophosphate vibrational modes characteristic of sulfide argyrodites in Raman spectroscopy of $Li_6PS_5(CN)_{1-x}Br_x$. As shown in Figure S8, the expected Raman-active modes of the PS_4^{3-} tetrahedra at 575, 420, and 265 cm^{-1} are observed for all members of the series.[5–12] We do not observe significant peak shifting as a function of x, suggesting that the PS_4^{3-} vibrational landscape is generally unchanged across the series (Figure S9). Li_6PS_5Br exhibits fluorescence under the Raman laser, which results in a rising background and poor signal-to-noise. Additionally, a small feature indicative of $P_2S_6^{4-}$ is present at 390 cm^{-1} in all spectra, which has previously been observed in low-temperature solution-phase and mechanochemical syntheses of Li_6PS_5Br.[12,13] Though we do not observe crystalline phases containing phosphorous or sulfide in the diffraction data, the presence of $P_2S_6^{4-}$ moieties may account for phosphorous and sulfur mass balance and

the diffuse scattering at low Q in the SXRD (Figure 2a).

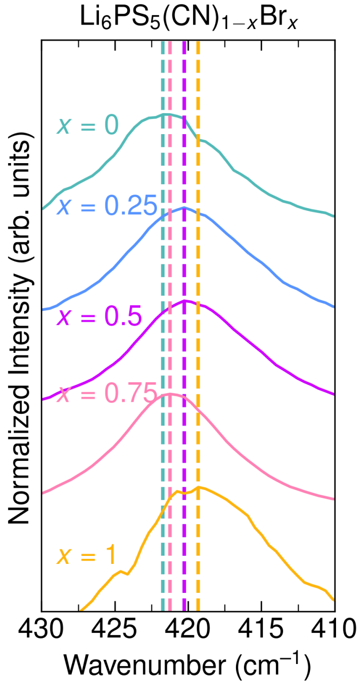

Figure S9: Baseline subtracted Raman spectra for $Li_6PS_5(CN)_{1-x}Br_x$. Dashed lines of each color indicate the wavenumber where the intensity is maximized for the PS_4^{3-} symmetric stretch.

Electrochemistry

Table S4: Ionic conductivity at 30 °C ($\sigma_{30°C}$), activation energy (E_A), and the temperature-independent Arrhenius prefactor (σ_0) for $Li_6PS_5(CN)_{1-x}Br_x$. Error represents the standard deviation between measurements for two pellets.

x	$\sigma_{30°C}$ (S cm^{-1})	E_A (meV)	σ_0 (K S cm^{-1})
0	1.56(4)E-4	471(2)	3.1(5)E6
0.25	1.5(3)E-4	432(6)	6.9(4)E5
0.5	2.37(3)E-4	435(9)	1.3(4)E6
0.75	4.0(4)E-4	416(3)	1.14(3)E6
1	1.289(1)E-4	443(8)	9.6(3)E5

Table S5: Parameters for R_1Q_1-Q_2 equivalent circuit fit of temperature-dependent EIS for Li_6PS_5CN, pellet 1. τ_1^{-1} represents the characteristic frequency of the R_1Q_1 circuit element. Parameters from three replicate frequency sweeps are included. Thickness = 2.139 mm.

T (°C)	#	R_1 (Ω)	σ (S cm^{-1})	n_1	Q_1(S/s^n)	C_1 (F)	τ_1^{-1} (Hz)	n_2	Q_2(S/s^n)
30	1	4.86E+03	1.56E-04	0.81	2.33E-10	9.54E-12	3.43E+06	0.81	1.67E-07
30	2	4.91E+03	1.54E-04	0.82	1.99E-10	9.52E-12	3.41E+06	0.81	1.71E-07
30	3	5.03E+03	1.50E-04	0.8	2.63E-10	9.46E-12	3.35E+06	0.82	1.59E-07
35	1	4.05E+03	1.87E-04	0.8	2.90E-10	9.35E-12	4.20E+06	0.83	1.51E-07
35	2	4.05E+03	1.87E-04	0.8	2.90E-10	9.36E-12	4.20E+06	0.83	1.51E-07
35	3	4.05E+03	1.87E-04	0.8	2.92E-10	9.37E-12	4.20E+06	0.83	1.51E-07
45	1	2.36E+03	3.21E-04	0.76	6.29E-10	9.16E-12	7.38E+06	0.86	1.27E-07
45	2	2.35E+03	3.22E-04	0.76	6.36E-10	9.18E-12	7.37E+06	0.86	1.27E-07
45	3	2.34E+03	3.23E-04	0.76	6.64E-10	9.01E-12	7.54E+06	0.86	1.26E-07
55	1	1.39E+03	5.45E-04	0.66	4.00E-09	6.90E-12	1.66E+07	0.89	1.14E-07
55	2	1.38E+03	5.47E-04	0.66	3.96E-09	6.95E-12	1.65E+07	0.89	1.14E-07
55	3	1.38E+03	5.49E-04	0.65	4.15E-09	6.91E-12	1.67E+07	0.89	1.14E-07
65	1	8.20E+02	9.23E-04	0.75	1.03E-09	9.71E-12	2.00E+07	0.85	8.10E-07
65	2	8.20E+02	9.23E-04	0.75	1.03E-09	9.70E-12	2.00E+07	0.85	8.11E-07
65	3	8.00E+02	9.46E-04	0.75	1.05E-09	9.95E-12	2.00E+07	0.85	9.00E-07
75	1	5.85E+02	1.29E-03	0.6	1.44E-08	5.94E-12	4.58E+07	0.9	1.20E-07
75	2	5.85E+02	1.29E-03	0.6	1.44E-08	5.94E-12	4.58E+07	0.9	1.20E-07
75	3	5.85E+02	1.29E-03	0.6	1.44E-08	5.94E-12	4.58E+07	0.9	1.20E-07
85	1	3.94E+02	1.92E-03	0.6	1.71E-08	6.07E-12	6.65E+07	0.91	1.28E-07
85	2	3.94E+02	1.92E-03	0.6	1.71E-08	6.07E-12	6.65E+07	0.91	1.28E-07
85	3	3.94E+02	1.92E-03	0.6	1.71E-08	6.07E-12	6.65E+07	0.91	1.28E-07
95	1	2.80E+02	2.70E-03	0.6	1.55E-08	4.14E-12	1.37E+08	0.9	1.68E-07
95	2	2.80E+02	2.70E-03	0.6	1.55E-08	4.14E-12	1.37E+08	0.9	1.68E-07
95	3	2.79E+02	2.71E-03	0.6	1.67E-08	4.68E-12	1.22E+08	0.9	1.68E-07

Table S6: Parameters for R_1Q_1-Q_2 equivalent circuit fit of temperature-dependent EIS for Li_6PS_5CN, pellet 2. τ_1^{-1} represents the characteristic frequency of the R_1Q_1 circuit element. Parameters from three replicate frequency sweeps are included. At 75 °C, only the last frequency sweep is included due to high variability. Thickness = 2.184 mm.

T (°C)	#	R_1 (Ω)	σ (S cm^{-1})	n_1	Q_1(S/s^n)	C_1 (F)	τ_1^{-1} (Hz)	n_2	Q_2(S/s^n)
30	1	4.82E+03	1.60E-04	0.83	1.57E-10	9.43E-12	3.50E+06	0.81	1.89E-07
30	2	4.85E+03	1.59E-04	0.83	1.55E-10	9.40E-12	3.49E+06	0.81	1.88E-07
30	3	4.87E+03	1.59E-04	0.83	1.60E-10	9.39E-12	3.48E+06	0.81	1.88E-07
35	1	3.77E+03	2.05E-04	0.81	2.74E-10	9.86E-12	4.28E+06	0.86	1.24E-07
35	2	3.77E+03	2.05E-04	0.81	2.74E-10	9.86E-12	4.28E+06	0.86	1.24E-07
35	3	3.77E+03	2.05E-04	0.81	2.73E-10	9.85E-12	4.29E+06	0.86	1.24E-07
45	1	2.19E+03	3.53E-04	0.75	6.56E-10	8.29E-12	8.78E+06	0.88	1.15E-07
45	2	2.19E+03	3.53E-04	0.76	6.29E-10	8.23E-12	8.84E+06	0.88	1.15E-07
45	3	2.20E+03	3.51E-04	0.75	6.23E-10	7.66E-12	9.43E+06	0.88	1.16E-07
55	1	1.31E+03	5.89E-04	0.67	2.62E-09	5.80E-12	2.09E+07	0.9	1.10E-07
55	2	1.29E+03	6.00E-04	0.65	4.18E-09	5.56E-12	2.22E+07	0.9	1.08E-07
55	3	1.28E+03	6.05E-04	0.65	4.01E-09	5.77E-12	2.16E+07	0.9	1.09E-07
65	1	7.43E+02	1.04E-03	0.75	8.37E-10	7.14E-12	3.00E+07	0.87	8.11E-07
65	2	7.40E+02	1.04E-03	0.75	8.37E-10	7.17E-12	3.00E+07	0.87	9.00E-07
65	3	7.43E+02	1.04E-03	0.75	8.37E-10	7.14E-12	3.00E+07	0.87	8.11E-07
75	1	5.60E+02	1.38E-03	0.75	7.57E-10	5.68E-12	5.00E+07	0.87	8.11E-07
85	1	3.58E+02	2.16E-03	0.6	1.36E-08	3.89E-12	1.14E+08	0.9	1.59E-07
85	2	3.58E+02	2.16E-03	0.6	1.36E-08	3.89E-12	1.14E+08	0.9	1.59E-07
85	3	3.58E+02	2.16E-03	0.6	1.36E-08	3.89E-12	1.14E+08	0.9	1.59E-07
95	1	2.78E+02	2.78E-03	0.6	1.29E-08	3.03E-12	1.88E+08	0.88	2.13E-07
95	2	2.78E+02	2.78E-03	0.6	1.29E-08	3.03E-12	1.88E+08	0.88	2.13E-07
95	3	2.73E+02	2.83E-03	0.6	1.21E-08	2.67E-12	2.18E+08	0.88	2.16E-07

Table S7: Parameters for R_1Q_1-Q_2 equivalent circuit fit of temperature-dependent EIS for $Li_6PS_5(CN)_{0.75}Br_{0.25}$, pellet 1. τ_1^{-1} represents the characteristic frequency of the R_1Q_1 circuit element. Parameters from three replicate frequency sweeps are included. Thickness = 2.113 mm.

T (°C)	#	R_1 (Ω)	σ (S cm^{-1})	n_1	Q_1(S/s^n)	C_1 (F)	τ_1^{-1} (Hz)	n_2	Q_2(S/s^n)
30	1	4.37E+03	1.71E-04	0.67	2.18E-09	7.24E-12	5.04E+06	0.76	1.90E-07
30	2	4.36E+03	1.71E-04	0.67	2.18E-09	7.22E-12	5.05E+06	0.76	1.91E-07
30	3	4.36E+03	1.71E-04	0.67	2.18E-09	7.22E-12	5.05E+06	0.76	1.91E-07
35	1	3.33E+03	2.25E-04	0.6	7.33E-09	6.41E-12	7.46E+06	0.8	1.43E-07
35	2	3.33E+03	2.25E-04	0.6	7.33E-09	6.41E-12	7.46E+06	0.8	1.43E-07
35	3	3.32E+03	2.25E-04	0.6	7.50E-09	6.42E-12	7.47E+06	0.8	1.43E-07
45	1	2.11E+03	3.54E-04	0.6	8.19E-09	5.48E-12	1.37E+07	0.83	1.26E-07
45	2	2.11E+03	3.54E-04	0.6	8.19E-09	5.48E-12	1.37E+07	0.83	1.26E-07
45	3	2.11E+03	3.55E-04	0.6	8.39E-09	5.69E-12	1.33E+07	0.83	1.26E-07
55	1	1.31E+03	5.70E-04	0.6	6.95E-09	3.04E-12	4.00E+07	0.79	4.05E-07
55	2	1.31E+03	5.70E-04	0.6	6.96E-09	3.04E-12	4.00E+07	0.79	4.05E-07
55	3	1.31E+03	5.70E-04	0.6	6.96E-09	3.04E-12	4.00E+07	0.79	4.05E-07
65	1	8.95E+02	8.35E-04	0.6	1.02E-08	4.45E-12	4.00E+07	0.79	4.50E-07
65	2	8.95E+02	8.35E-04	0.6	1.02E-08	4.45E-12	4.00E+07	0.79	4.50E-07
65	3	8.95E+02	8.35E-04	0.6	1.02E-08	4.45E-12	4.00E+07	0.79	4.50E-07
65	1	6.00E+02	1.25E-03	0.6	1.33E-08	5.31E-12	5.00E+07	0.77	4.50E-07
75	2	6.00E+02	1.25E-03	0.6	1.33E-08	5.31E-12	5.00E+07	0.77	4.50E-07
75	3	5.99E+02	1.25E-03	0.6	1.33E-08	5.31E-12	5.00E+07	0.77	4.50E-07
85	1	4.40E+02	1.70E-03	0.6	1.27E-08	4.02E-12	9.00E+07	0.8	6.31E-07
85	2	4.40E+02	1.70E-03	0.6	1.27E-08	4.02E-12	9.00E+07	0.8	6.30E-07
85	3	4.40E+02	1.70E-03	0.6	1.27E-08	4.02E-12	9.00E+07	0.8	6.30E-07
95	1	2.80E+02	2.67E-03	0.6	1.88E-08	5.68E-12	1.00E+08	0.8	7.00E-07
95	2	2.80E+02	2.67E-03	0.6	1.88E-08	5.68E-12	1.00E+08	0.8	7.00E-07
95	3	2.80E+02	2.67E-03	0.6	1.88E-08	5.68E-12	1.00E+08	0.8	7.00E-07

Table S8: Parameters for R_1Q_1-Q_2 equivalent circuit fit of temperature-dependent EIS for $Li_6PS_5CN_{0.75}Br_{0.25}$, pellet 2. τ_1^{-1} represents the characteristic frequency of the R_1Q_1 circuit element. Parameters from three replicate frequency sweeps are included. At 35 °C, only the last frequency sweep is included due to high variability. Thickness = 2.163 mm.

T (°C)	#	R_1 (Ω)	σ (S cm^{-1})	n_1	Q_1(S/s^n)	C_1 (F)	τ_1^{-1} (Hz)	n_2	Q_2(S/s^n)
30	1	5.94E+03	1.29E-04	0.61	4.78E-09	6.29E-12	4.26E+06	0.81	1.64E-07
30	2	6.02E+03	1.27E-04	0.6	5.12E-09	5.61E-12	4.72E+06	0.82	1.48E-07
30	3	5.94E+03	1.29E-04	0.62	4.41E-09	6.28E-12	4.26E+06	0.83	1.46E-07
35	1	4.27E+03	1.79E-04	0.62	3.93E-09	5.35E-12	6.96E+06	0.8	2.00E-07
45	1	2.82E+03	2.71E-04	0.6	7.18E-09	5.33E-12	1.06E+07	0.86	1.24E-07
45	2	2.82E+03	2.71E-04	0.6	7.18E-09	5.33E-12	1.06E+07	0.86	1.24E-07
45	3	2.82E+03	2.71E-04	0.6	7.18E-09	5.33E-12	1.06E+07	0.86	1.24E-07
55	1	1.70E+03	4.49E-04	0.61	4.26E-09	2.33E-12	4.00E+07	0.8	4.05E-07
55	2	1.71E+03	4.48E-04	0.6	5.33E-09	2.33E-12	4.00E+07	0.8	4.05E-07
55	3	1.69E+03	4.52E-04	0.64	2.69E-09	2.35E-12	4.00E+07	0.8	4.06E-07
65	1	1.17E+03	6.54E-04	0.6	6.77E-09	2.72E-12	5.00E+07	0.85	4.05E-07
65	2	1.17E+03	6.54E-04	0.6	6.75E-09	2.72E-12	5.00E+07	0.85	4.05E-07
65	3	1.17E+03	6.54E-04	0.6	6.82E-09	2.72E-12	5.00E+07	0.85	4.05E-07
75	1	8.05E+02	9.50E-04	0.6	9.91E-09	3.95E-12	5.00E+07	0.83	4.50E-07
75	2	8.05E+02	9.50E-04	0.6	9.91E-09	3.95E-12	5.00E+07	0.83	4.50E-07
75	3	8.05E+02	9.50E-04	0.6	9.89E-09	3.95E-12	5.00E+07	0.83	4.50E-07
85	1	5.10E+02	1.50E-03	0.6	1.10E-08	3.47E-12	9.00E+07	0.85	6.30E-07
85	2	5.05E+02	1.51E-03	0.6	1.11E-08	3.50E-12	9.00E+07	0.85	7.00E-07
85	3	5.05E+02	1.51E-03	0.6	1.11E-08	3.50E-12	9.00E+07	0.85	7.00E-07
95	1	3.50E+02	2.19E-03	0.6	9.92E-09	2.27E-12	2.00E+08	0.85	6.30E-07
95	2	3.50E+02	2.19E-03	0.6	9.92E-09	2.27E-12	2.00E+08	0.85	6.30E-07
95	3	3.50E+02	2.19E-03	0.6	9.92E-09	2.27E-12	2.00E+08	0.85	6.30E-07

Table S9: Parameters for R_1Q_1-Q_2 equivalent circuit fit of temperature-dependent EIS for $Li_6PS_5CN_{0.5}Br_{0.5}$, pellet 1. τ_1^{-1} represents the characteristic frequency of the R_1Q_1 circuit element. Parameters from three replicate frequency sweeps are included. At 45 °C, only the last frequency sweep is included due to high variability. Thickness = 2.057 mm.

T (°C)	#	R_1 (Ω)	σ (S cm^{-1})	n_1	Q_1(S/s^n)	C_1 (F)	τ_1^{-1} (Hz)	n_2	Q_2(S/s^n)
30	1	3.10E+03	2.35E-04	0.83	1.73E-10	9.16E-12	5.62E+06	0.88	1.14E-07
30	2	3.09E+03	2.35E-04	0.83	1.70E-10	9.34E-12	5.51E+06	0.88	1.15E-07
30	3	3.09E+03	2.35E-04	0.84	1.50E-10	9.42E-12	5.47E+06	0.88	1.14E-07
35	1	2.32E+03	3.14E-04	0.82	2.07E-10	8.73E-12	7.87E+06	0.9	9.87E-08
35	2	2.32E+03	3.14E-04	0.81	2.40E-10	9.01E-12	7.63E+06	0.9	9.65E-08
35	3	2.31E+03	3.15E-04	0.82	2.31E-10	8.71E-12	7.91E+06	0.9	9.66E-08
45	1	1.64E+03	4.43E-04	0.72	8.49E-10	4.18E-12	2.32E+07	0.91	9.47E-08
55	1	8.89E+02	8.19E-04	0.67	2.28E-09	3.73E-12	4.81E+07	0.92	9.53E-08
55	2	8.84E+02	8.23E-04	0.67	2.22E-09	3.98E-12	4.53E+07	0.92	9.48E-08
55	3	8.84E+02	8.23E-04	0.71	1.24E-09	4.28E-12	4.20E+07	0.92	9.57E-08
65	1	5.92E+02	1.23E-03	0.67	2.10E-09	2.83E-12	9.49E+07	0.93	9.93E-08
65	2	5.91E+02	1.23E-03	0.67	2.22E-09	2.90E-12	9.29E+07	0.93	9.94E-08
65	3	5.91E+02	1.23E-03	0.66	2.58E-09	2.94E-12	9.15E+07	0.93	9.95E-08
75	1	3.85E+02	1.89E-03	0.67	3.45E-09	4.13E-12	1.00E+08	0.93	1.04E-07
75	2	3.85E+02	1.89E-03	0.67	3.45E-09	4.13E-12	1.00E+08	0.93	1.04E-07
75	3	3.85E+02	1.89E-03	0.67	3.45E-09	4.13E-12	1.00E+08	0.93	1.04E-07
85	1	3.07E+02	2.37E-03	0.83	1.61E-10	5.19E-12	1.00E+08	0.92	1.17E-07
85	2	3.07E+02	2.37E-03	0.83	1.53E-10	5.19E-12	1.00E+08	0.92	1.18E-07
85	3	3.07E+02	2.37E-03	0.82	1.88E-10	5.18E-12	1.00E+08	0.92	1.17E-07
95	1	2.09E+02	3.48E-03	0.77	7.87E-10	7.61E-12	1.00E+08	0.91	1.49E-07
95	2	2.09E+02	3.49E-03	0.79	5.68E-10	7.62E-12	1.00E+08	0.91	1.50E-07
95	3	2.08E+02	3.50E-03	0.78	7.13E-10	7.67E-12	1.00E+08	0.91	1.50E-07

Table S10: Parameters for R_1Q_1-Q_2 equivalent circuit fit of temperature-dependent EIS for $Li_6PS_5CN_{0.5}Br_{0.5}$, pellet 2. τ_1^{-1} represents the characteristic frequency of the R_1Q_1 circuit element. Parameters from three replicate frequency sweeps are included. At 35 °C, only the last frequency sweep is included due to high variability. Thickness = 2.068 mm.

T (°C)	#	R_1 (Ω)	σ (S cm^{-1})	n_1	Q_1(S/s^n)	C_1 (F)	τ_1^{-1} (Hz)	n_2	Q_2(S/s^n)
30	1	3.07E+03	2.39E-04	0.8	2.68E-10	7.53E-12	6.90E+06	0.9	7.24E-08
30	2	3.06E+03	2.39E-04	0.79	2.87E-10	7.40E-12	7.03E+06	0.9	7.19E-08
30	3	3.06E+03	2.39E-04	0.79	2.87E-10	7.40E-12	7.03E+06	0.9	7.19E-08
35	1	2.43E+03	3.01E-04	0.83	1.37E-10	6.26E-12	1.04E+07	0.91	6.78E-08
45	1	1.44E+03	5.09E-04	0.73	7.64E-10	4.27E-12	2.59E+07	0.93	6.52E-08
45	2	1.46E+03	5.02E-04	0.73	6.74E-10	3.93E-12	2.78E+07	0.92	6.75E-08
45	3	1.41E+03	5.20E-04	0.69	1.50E-09	4.05E-12	2.79E+07	0.93	6.34E-08
55	1	8.76E+02	8.34E-04	0.6	7.68E-09	2.74E-12	6.63E+07	0.94	6.46E-08
55	2	8.76E+02	8.34E-04	0.6	7.68E-09	2.74E-12	6.63E+07	0.94	6.46E-08
55	3	8.76E+02	8.34E-04	0.6	7.68E-09	2.74E-12	6.63E+07	0.94	6.46E-08
65	1	5.80E+02	1.26E-03	0.6	8.80E-09	2.74E-12	1.00E+08	0.94	6.75E-08
65	2	5.80E+02	1.26E-03	0.6	8.80E-09	2.74E-12	1.00E+08	0.94	6.75E-08
65	3	5.80E+02	1.26E-03	0.6	8.80E-09	2.74E-12	1.00E+08	0.94	6.75E-08
75	1	3.94E+02	1.86E-03	0.92	4.08E-11	9.23E-12	4.38E+07	0.94	7.39E-08
75	2	3.94E+02	1.86E-03	0.92	4.42E-11	9.17E-12	4.41E+07	0.94	7.40E-08
75	3	3.94E+02	1.86E-03	0.92	4.43E-11	9.11E-12	4.44E+07	0.94	7.41E-08
85	1	2.95E+02	2.48E-03	0.8	1.02E-10	1.35E-12	4.00E+08	0.91	6.30E-07
85	2	2.95E+02	2.48E-03	0.8	1.02E-10	1.35E-12	4.00E+08	0.91	6.30E-07
85	3	2.95E+02	2.48E-03	0.87	2.26E-11	1.35E-12	4.00E+08	0.91	6.30E-07
95	1	2.09E+02	3.49E-03	0.83	2.45E-10	7.60E-12	1.00E+08	0.93	9.69E-08
95	2	2.09E+02	3.49E-03	0.83	2.45E-10	7.60E-12	1.00E+08	0.93	9.69E-08
95	3	2.09E+02	3.49E-03	0.83	2.45E-10	7.60E-12	1.00E+08	0.93	9.69E-08

Table S11: Parameters for R_1Q_1-Q_2 equivalent circuit fit of temperature-dependent EIS for $Li_6PS_5CN_{0.25}Br_{0.75}$, pellet 1. τ_1^{-1} represents the characteristic frequency of the R_1Q_1 circuit element. Parameters from three replicate frequency sweeps are included. Thickness = 1.979 mm.

T (°C)	#	R_1 (Ω)	σ (S cm^{-1})	n_1	Q_1(S/s^n)	C_1 (F)	τ_1^{-1} (Hz)	n_2	Q_2(S/s^n)
30	1	1.87E+03	3.74E-04	0.68	1.99E-09	4.96E-12	1.71E+07	0.85	1.19E-07
30	2	1.87E+03	3.74E-04	0.67	2.24E-09	5.23E-12	1.63E+07	0.85	1.19E-07
30	3	1.87E+03	3.75E-04	0.67	2.15E-09	5.18E-12	1.65E+07	0.85	1.19E-07
35	1	1.44E+03	4.86E-04	0.6	8.54E-09	4.55E-12	2.43E+07	0.87	9.74E-08
35	2	1.44E+03	4.86E-04	0.6	8.54E-09	4.55E-12	2.43E+07	0.87	9.74E-08
35	3	1.44E+03	4.86E-04	0.6	8.54E-09	4.55E-12	2.43E+07	0.87	9.74E-08
45	1	7.75E+02	9.03E-04	0.85	1.68E-10	1.03E-11	2.00E+07	0.83	1.80E-07
45	2	7.75E+02	9.03E-04	0.85	1.68E-10	1.03E-11	2.00E+07	0.83	1.80E-07
45	3	7.75E+02	9.03E-04	0.85	1.68E-10	1.03E-11	2.00E+07	0.83	1.80E-07
55	1	4.65E+02	1.51E-03	1	6.84E-12	6.84E-12	5.00E+07	0.82	2.00E-07
55	2	4.65E+02	1.51E-03	0.9	4.73E-11	6.85E-12	5.00E+07	0.83	1.80E-07
55	3	4.65E+02	1.51E-03	1	6.93E-12	6.85E-12	5.00E+07	0.82	1.99E-07
65	1	3.05E+02	2.29E-03	0.92	3.56E-11	6.52E-12	8.00E+07	0.84	1.80E-07
65	2	3.05E+02	2.29E-03	0.86	1.07E-10	6.52E-12	8.00E+07	0.82	2.19E-07
65	3	3.05E+02	2.29E-03	0.85	1.31E-10	6.52E-12	8.00E+07	0.84	1.80E-07
75	1	2.04E+02	3.43E-03	0.94	3.55E-11	9.75E-12	8.00E+07	0.85	1.81E-07
75	2	2.04E+02	3.43E-03	1	1.07E-11	9.75E-12	8.00E+07	0.84	1.92E-07
75	3	2.04E+02	3.43E-03	0.87	1.27E-10	9.75E-12	8.00E+07	0.85	1.80E-07
85	1	1.92E+02	3.65E-03	1	9.12E-12	8.29E-12	1.00E+08	0.85	1.91E-07
85	2	1.92E+02	3.65E-03	0.95	2.29E-11	8.29E-12	1.00E+08	0.85	1.95E-07
95	1	1.31E+02	5.34E-03	0.85	1.36E-10	6.07E-12	2.00E+08	0.85	2.19E-07
95	2	1.31E+02	5.34E-03	1	6.12E-12	6.07E-12	2.00E+08	0.85	2.17E-07
95	3	1.31E+02	5.34E-03	0.87	9.89E-11	6.07E-12	2.00E+08	0.85	2.18E-07

Table S12: Parameters for R_1Q_1-Q_2 equivalent circuit fit of temperature-dependent EIS for $Li_6PS_5CN_{0.25}Br_{0.75}$, pellet 2. τ_1^{-1} represents the characteristic frequency of the R_1Q_1 circuit element. Parameters from three replicate frequency sweeps are included. Thickness = 2.059 mm.

T (°C)	#	R_1 (Ω)	σ (S cm^{-1})	n_1	Q_1(S/s^n)	C_1 (F)	τ_1^{-1} (Hz)	n_2	Q_2(S/s^n)
30	1	1.69E+03	4.32E-04	0.86	8.88E-11	6.75E-12	1.40E+07	0.86	1.39E-07
30	2	1.68E+03	4.33E-04	0.86	8.81E-11	6.87E-12	1.38E+07	0.87	1.35E-07
30	3	1.68E+03	4.33E-04	0.86	8.81E-11	6.87E-12	1.38E+07	0.87	1.35E-07
35	1	1.27E+03	5.75E-04	0.75	5.53E-10	4.94E-12	2.54E+07	0.89	1.11E-07
35	2	1.26E+03	5.76E-04	0.75	5.39E-10	5.04E-12	2.49E+07	0.89	1.11E-07
35	3	1.26E+03	5.77E-04	0.76	4.96E-10	5.14E-12	2.45E+07	0.89	1.11E-07
45	1	7.15E+02	1.02E-03	0.85	1.29E-10	7.42E-12	3.00E+07	0.87	1.80E-07
45	2	7.15E+02	1.02E-03	0.85	1.29E-10	7.42E-12	3.00E+07	0.87	1.80E-07
45	3	7.15E+02	1.02E-03	0.85	1.29E-10	7.42E-12	3.00E+07	0.87	1.80E-07
55	1	4.71E+02	1.55E-03	0.62	7.75E-09	3.38E-12	1.00E+08	0.91	1.12E-07
55	2	4.71E+02	1.55E-03	0.62	7.75E-09	3.38E-12	1.00E+08	0.91	1.12E-07
55	3	4.90E+02	1.49E-03	0.71	1.16E-09	3.28E-12	9.90E+07	0.91	1.14E-07
65	1	3.03E+02	2.41E-03	0.76	6.27E-10	5.26E-12	1.00E+08	0.91	1.15E-07
65	2	3.03E+02	2.41E-03	0.76	6.27E-10	5.26E-12	1.00E+08	0.91	1.15E-07
65	3	3.03E+02	2.41E-03	0.76	6.27E-10	5.26E-12	1.00E+08	0.91	1.15E-07
75	1	2.29E+02	3.17E-03	0.82	2.46E-10	6.95E-12	9.99E+07	0.92	1.18E-07
75	2	2.29E+02	3.17E-03	0.82	2.46E-10	6.95E-12	9.99E+07	0.92	1.18E-07
75	3	2.29E+02	3.17E-03	0.82	2.46E-10	6.95E-12	9.99E+07	0.92	1.18E-07
85	1	1.53E+02	4.76E-03	0.98	1.45E-11	1.04E-11	1.00E+08	0.87	2.01E-07
85	2	1.53E+02	4.76E-03	0.94	3.79E-11	1.04E-11	1.00E+08	0.88	1.81E-07
85	3	1.53E+02	4.76E-03	0.99	1.19E-11	1.04E-11	1.00E+08	0.88	1.80E-07
95	1	1.27E+02	5.76E-03	0.99	2.23E-11	1.77E-11	7.11E+07	0.91	1.45E-07
95	2	1.26E+02	5.77E-03	1	1.33E-11	1.33E-11	9.45E+07	0.91	1.45E-07
95	3	1.26E+02	5.77E-03	1	1.33E-11	1.33E-11	9.45E+07	0.91	1.45E-07

Table S13: Parameters for R_1Q_1-Q_2 equivalent circuit fit of temperature-dependent EIS for Li_6PS_5Br, pellet 1. τ_1^{-1} represents the characteristic frequency of the R_1Q_1 circuit element. Parameters from three replicate frequency sweeps are included. Thickness = 1.851 mm.

T (°C)	#	R_1 (Ω)	σ (S cm^{-1})	n_1	Q_1(S/s^n)	C_1 (F)	τ_1^{-1} (Hz)	n_2	Q_2(S/s^n)
30	1	5.08E+03	1.29E-04	0.79	4.12E-10	1.39E-11	2.25E+06	0.85	1.63E-07
30	2	5.08E+03	1.29E-04	0.79	4.12E-10	1.39E-11	2.25E+06	0.85	1.63E-07
30	3	5.08E+03	1.29E-04	0.79	4.13E-10	1.39E-11	2.25E+06	0.85	1.63E-07
35	1	3.73E+03	1.76E-04	0.78	5.85E-10	1.39E-11	3.07E+06	0.88	1.43E-07
35	2	3.73E+03	1.76E-04	0.78	5.80E-10	1.39E-11	3.07E+06	0.88	1.43E-07
35	3	3.73E+03	1.76E-04	0.78	5.80E-10	1.39E-11	3.07E+06	0.88	1.43E-07
45	1	2.19E+03	2.98E-04	0.77	6.92E-10	1.37E-11	5.30E+06	0.89	1.33E-07
45	2	2.19E+03	2.99E-04	0.77	7.62E-10	1.35E-11	5.40E+06	0.9	1.33E-07
45	3	2.19E+03	2.99E-04	0.77	7.74E-10	1.35E-11	5.40E+06	0.9	1.33E-07
55	1	1.36E+03	4.80E-04	0.74	1.26E-09	1.28E-11	9.13E+06	0.91	1.31E-07
55	2	1.36E+03	4.82E-04	0.74	1.30E-09	1.28E-11	9.17E+06	0.91	1.32E-07
55	3	1.36E+03	4.83E-04	0.73	1.47E-09	1.25E-11	9.41E+06	0.91	1.31E-07
65	1	8.50E+02	7.70E-04	0.75	1.23E-09	1.25E-11	1.50E+07	0.87	8.10E-07
65	2	8.50E+02	7.70E-04	0.75	1.20E-09	1.25E-11	1.50E+07	0.87	8.10E-07
65	3	8.30E+02	7.89E-04	0.75	1.26E-09	1.28E-11	1.50E+07	0.87	9.00E-07
75	1	6.10E+02	1.07E-03	0.75	1.17E-09	1.04E-11	2.50E+07	0.89	8.11E-07
75	2	6.10E+02	1.07E-03	0.75	1.17E-09	1.04E-11	2.50E+07	0.89	8.12E-07
75	3	6.10E+02	1.07E-03	0.75	1.17E-09	1.04E-11	2.50E+07	0.89	8.10E-07
85	1	4.57E+02	1.43E-03	0.6	1.87E-08	7.80E-12	4.47E+07	0.91	1.80E-07
85	2	4.57E+02	1.43E-03	0.6	1.87E-08	7.80E-12	4.47E+07	0.91	1.80E-07
85	3	4.54E+02	1.44E-03	0.6	1.65E-08	6.44E-12	5.44E+07	0.91	1.81E-07
95	1	2.93E+02	2.23E-03	0.6	1.97E-08	6.37E-12	8.52E+07	0.88	3.07E-07
95	2	2.88E+02	2.28E-03	0.6	1.83E-08	5.53E-12	1.00E+08	0.88	3.17E-07
95	3	2.88E+02	2.28E-03	0.6	1.83E-08	5.53E-12	1.00E+08	0.88	3.17E-07

Table S14: Parameters for R_1Q_1-Q_2 equivalent circuit fit of temperature-dependent EIS for Li_6PS_5Br, pellet 2. τ_1^{-1} represents the characteristic frequency of the R_1Q_1 circuit element. Parameters from three replicate frequency sweeps are included. Thickness = 2.044 mm.

T (°C)	#	R_1 (Ω)	σ (S cm^{-1})	n_1	Q_1(S/s^n)	C_1 (F)	τ_1^{-1} (Hz)	n_2	Q_2(S/s^n)
30	1	5.58E+03	1.29E-04	0.84	1.76E-10	1.20E-11	2.37E+06	0.76	2.72E-07
30	2	5.60E+03	1.29E-04	0.84	1.79E-10	1.21E-11	2.36E+06	0.76	2.70E-07
30	3	5.60E+03	1.29E-04	0.84	1.75E-10	1.21E-11	2.35E+06	0.76	2.69E-07
35	1	4.11E+03	1.76E-04	0.8	3.81E-10	1.24E-11	3.12E+06	0.83	1.61E-07
35	2	4.11E+03	1.76E-04	0.8	3.80E-10	1.24E-11	3.12E+06	0.83	1.61E-07
35	3	4.11E+03	1.76E-04	0.8	3.77E-10	1.25E-11	3.10E+06	0.83	1.65E-07
45	1	2.49E+03	2.90E-04	0.78	5.81E-10	1.23E-11	5.21E+06	0.85	1.56E-07
45	2	2.49E+03	2.91E-04	0.77	6.02E-10	1.22E-11	5.23E+06	0.85	1.57E-07
45	3	2.48E+03	2.91E-04	0.77	6.08E-10	1.22E-11	5.23E+06	0.85	1.57E-07
55	1	1.56E+03	4.62E-04	0.73	1.39E-09	1.11E-11	9.15E+06	0.87	1.55E-07
55	2	1.56E+03	4.62E-04	0.73	1.39E-09	1.11E-11	9.15E+06	0.87	1.55E-07
55	3	1.56E+03	4.63E-04	0.73	1.32E-09	1.10E-11	9.26E+06	0.87	1.55E-07
65	1	9.86E+02	7.33E-04	0.78	5.55E-10	1.01E-11	1.60E+07	0.85	2.08E-07
65	2	9.86E+02	7.33E-04	0.78	5.55E-10	1.01E-11	1.60E+07	0.85	2.08E-07
75	1	6.86E+02	1.05E-03	0.84	2.65E-10	1.36E-11	1.70E+07	0.84	2.20E-07
75	2	6.86E+02	1.05E-03	0.84	2.66E-10	1.36E-11	1.70E+07	0.84	2.14E-07
75	3	6.86E+02	1.05E-03	0.84	2.66E-10	1.36E-11	1.70E+07	0.84	2.14E-07
85	1	5.10E+02	1.42E-03	0.84	2.21E-10	1.04E-11	3.00E+07	0.84	2.19E-07
85	2	5.10E+02	1.42E-03	0.84	2.21E-10	1.04E-11	3.00E+07	0.84	2.19E-07
85	3	5.10E+02	1.42E-03	0.84	2.21E-10	1.04E-11	3.00E+07	0.84	2.19E-07
95	1	3.30E+02	2.19E-03	0.84	1.93E-10	8.04E-12	6.00E+07	0.84	2.20E-07
95	2	3.30E+02	2.19E-03	0.84	1.94E-10	8.04E-12	6.00E+07	0.84	2.15E-07
95	3	3.30E+02	2.19E-03	0.84	1.94E-10	8.04E-12	6.00E+07	0.84	2.19E-07

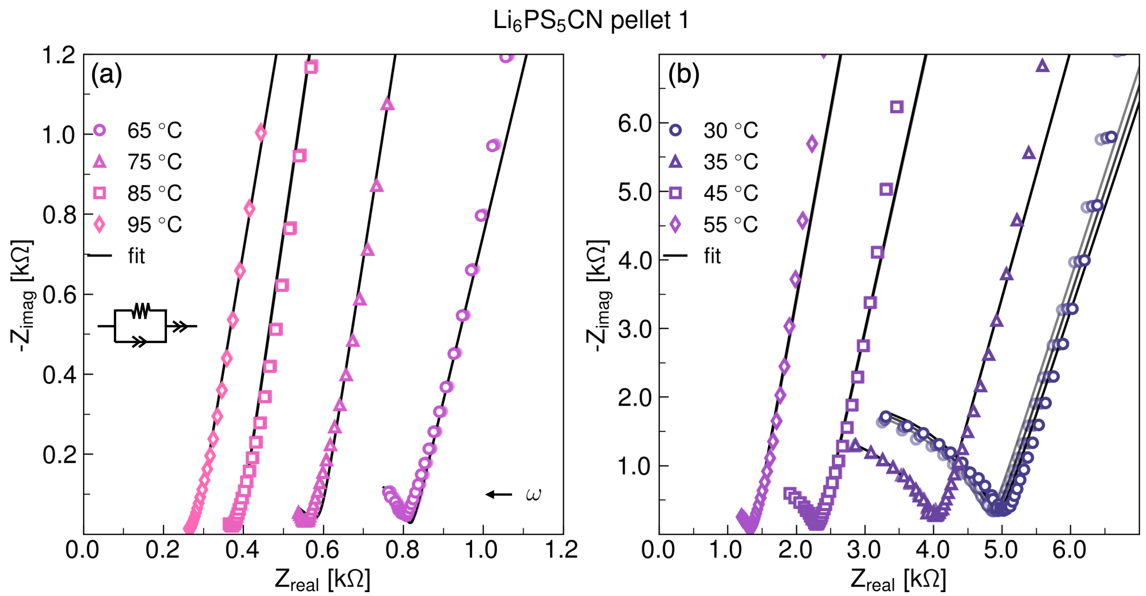

Figure S10: Temperature-dependent Nyquist plots of the last 3 replicate scans (decreasing transparency with later replicates) at each temperature for pellet 1 of Li_6PS_5CN. (a) High and (b) low temperature data have been separated for clarity. Data points are represented by markers, and the solid lines indicate the calculated fit to the $(R_1Q_1) + Q_2$ equivalent circuit model.

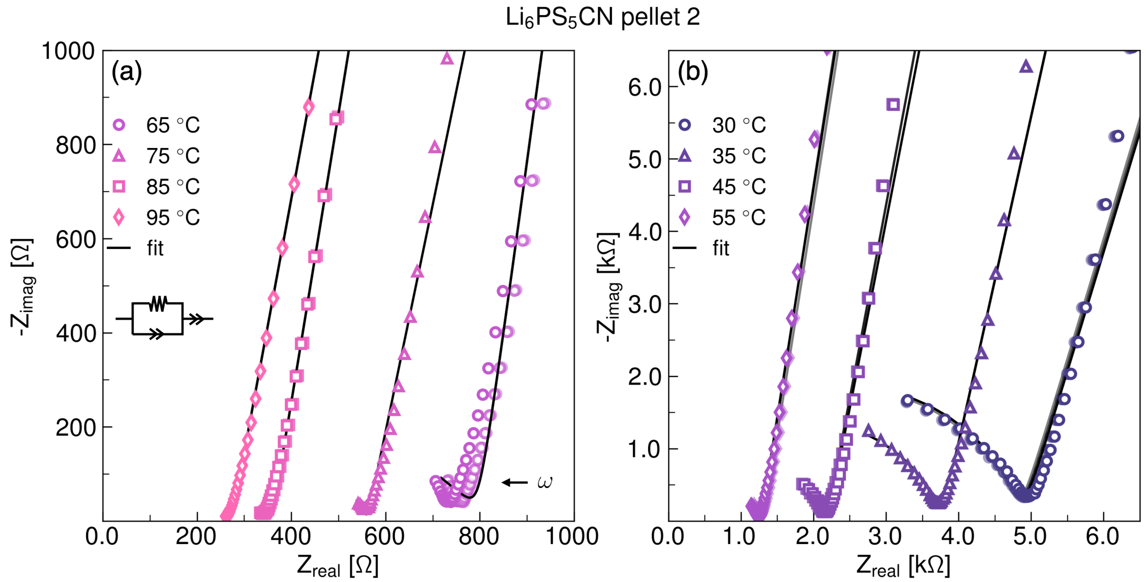

Figure S11: Temperature-dependent Nyquist plots of the last 3 replicate scans (decreasing transparency with later replicates) at each temperature for pellet 2 of Li_6PS_5CN. At 75 °C, only the last stable dataset is shown due to high variability. (a) High and (b) low temperature data have been separated for clarity. Data points are represented by markers, and the solid lines indicate the calculated fit to the $(R_1Q_1) + Q_2$ equivalent circuit model.

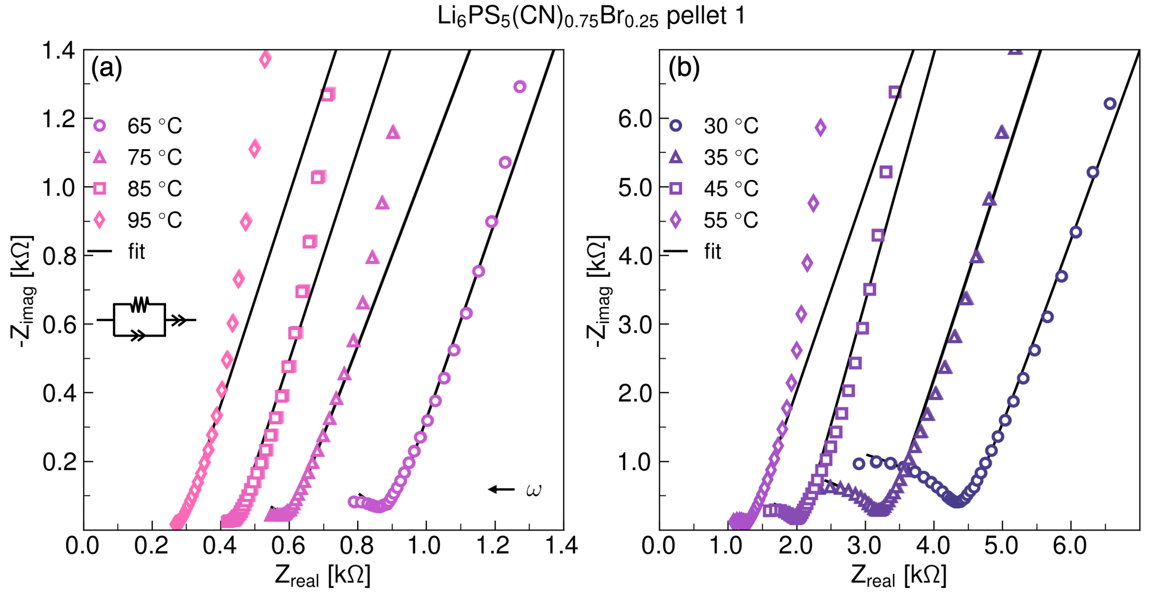

Figure S12: Temperature-dependent Nyquist plots of the last 3 replicate scans (decreasing transparency with later replicates) at each temperature for pellet 1 of $Li_6PS_5(CN)_{0.75}Br_{0.25}$. (a) High and (b) low temperature data have been separated for clarity. Data points are represented by markers, and the solid lines indicate the calculated fit to the $(R_1Q_1) + Q_2$ equivalent circuit model.

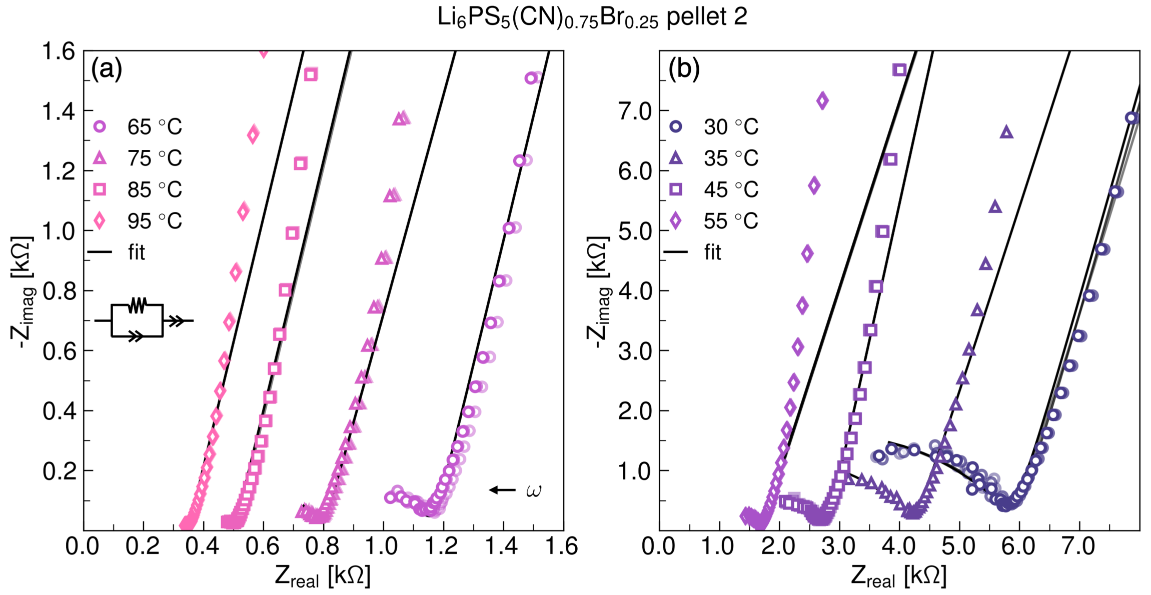

Figure S13: Temperature-dependent Nyquist plots of the last 3 replicate scans (decreasing transparency with later replicates) at each temperature for pellet 2 of $Li_6PS_5(CN)_{0.75}Br_{0.25}$. At 35 °C, only the last stable dataset is shown due to high variability. (a) High and (b) low temperature data have been separated for clarity. Data points are represented by markers, and the solid lines indicate the calculated fit to the $(R_1Q_1) + Q_2$ equivalent circuit model.

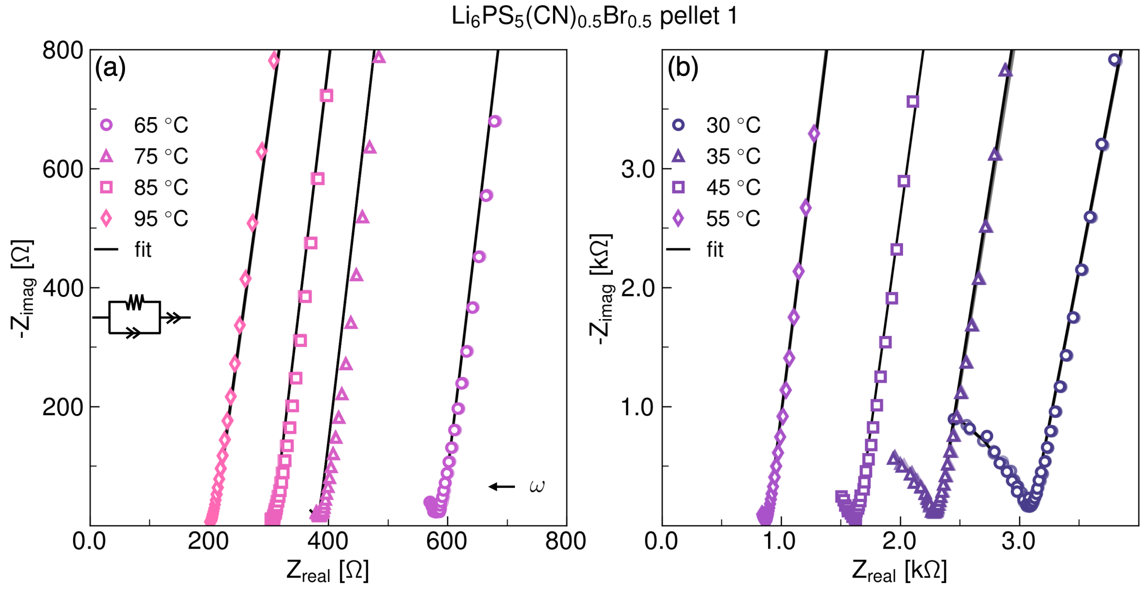

Figure S14: Temperature-dependent Nyquist plots of the last 3 replicate scans (decreasing transparency with later replicates) at each temperature for pellet 1 of $Li_6PS_5(CN)_{0.5}Br_{0.5}$. At 45 °C, only the last stable dataset is shown due to high variability. (a) High and (b) low temperature data have been separated for clarity. Data points are represented by markers, and the solid lines indicate the calculated fit to the $(R_1Q_1) + Q_2$ equivalent circuit model.

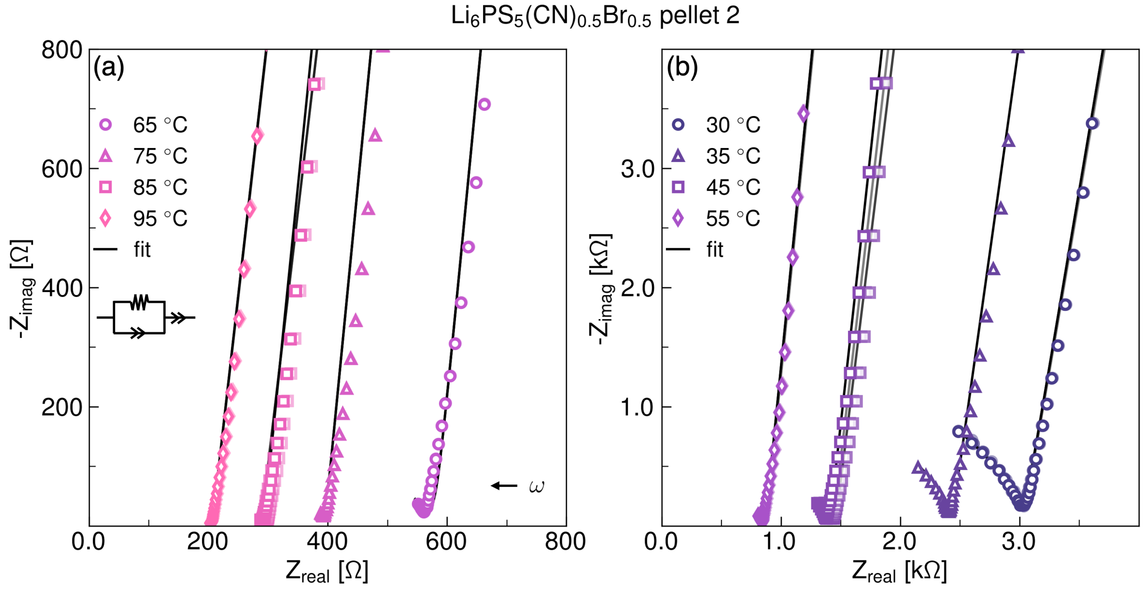

Figure S15: Temperature-dependent Nyquist plots of the last 3 replicate scans (decreasing transparency with later replicates) at each temperature for pellet 2 of $Li_6PS_5(CN)_{0.5}Br_{0.5}$. At 35 °C, only the last stable dataset is shown due to high variability. (a) High and (b) low temperature data have been separated for clarity. Data points are represented by markers, and the solid lines indicate the calculated fit to the $(R_1Q_1) + Q_2$ equivalent circuit model.

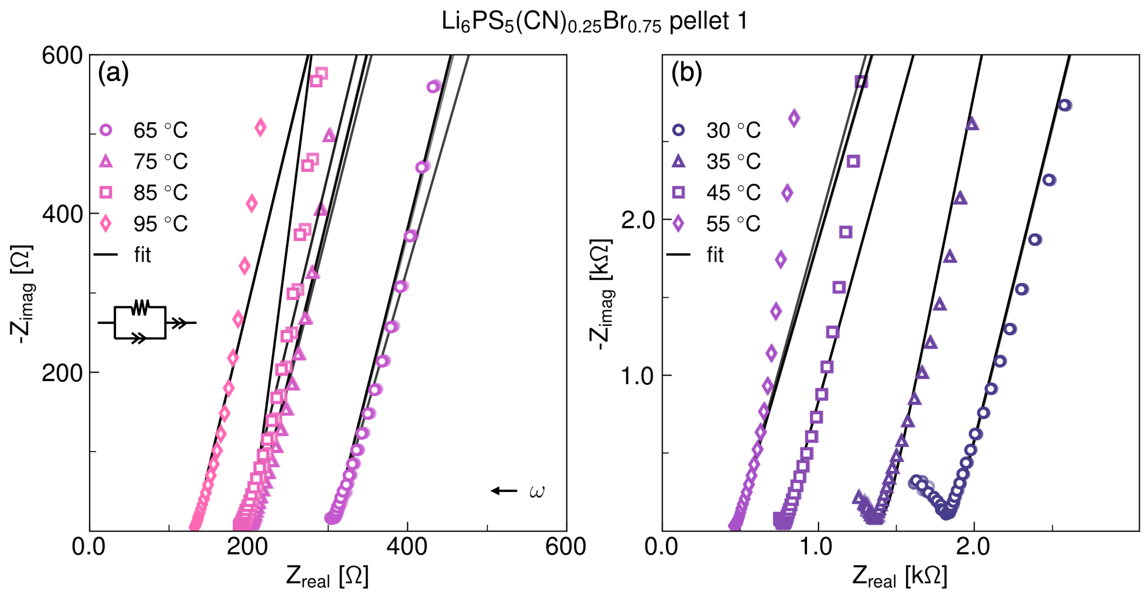

Figure S16: Temperature-dependent Nyquist plots of the last 3 replicate scans (decreasing transparency with later replicates) at each temperature for pellet 1 of $Li_6PS_5(CN)_{0.25}Br_{0.75}$. (a) High and (b) low temperature data have been separated for clarity. Data points are represented by markers, and the solid lines indicate the calculated fit to the $(R_1Q_1) + Q_2$ equivalent circuit model.

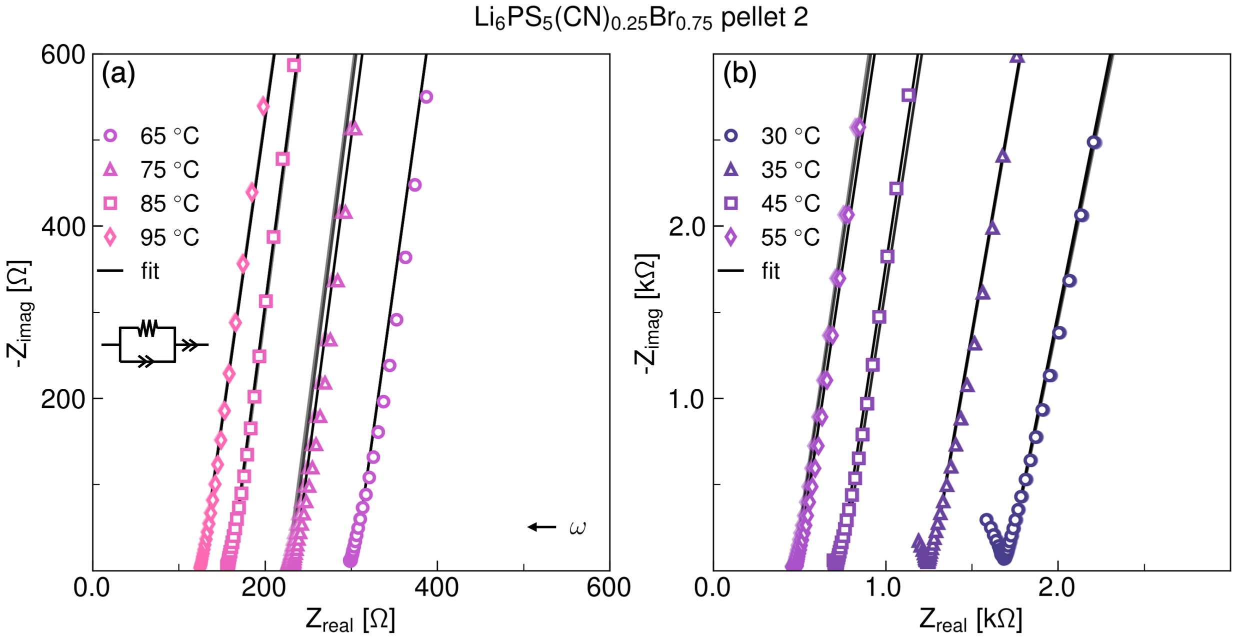

Figure S17: Temperature-dependent Nyquist plots of the last 3 replicate scans (decreasing transparency with later replicates) at each temperature for pellet 2 of $Li_6PS_5(CN)_{0.25}Br_{0.75}$. High (a) and low (b) temperature data are separated for clarity. Data points are represented by markers, and the solid lines indicate the calculated fit to the $(R_1Q_1) + Q_2$ equivalent circuit model.

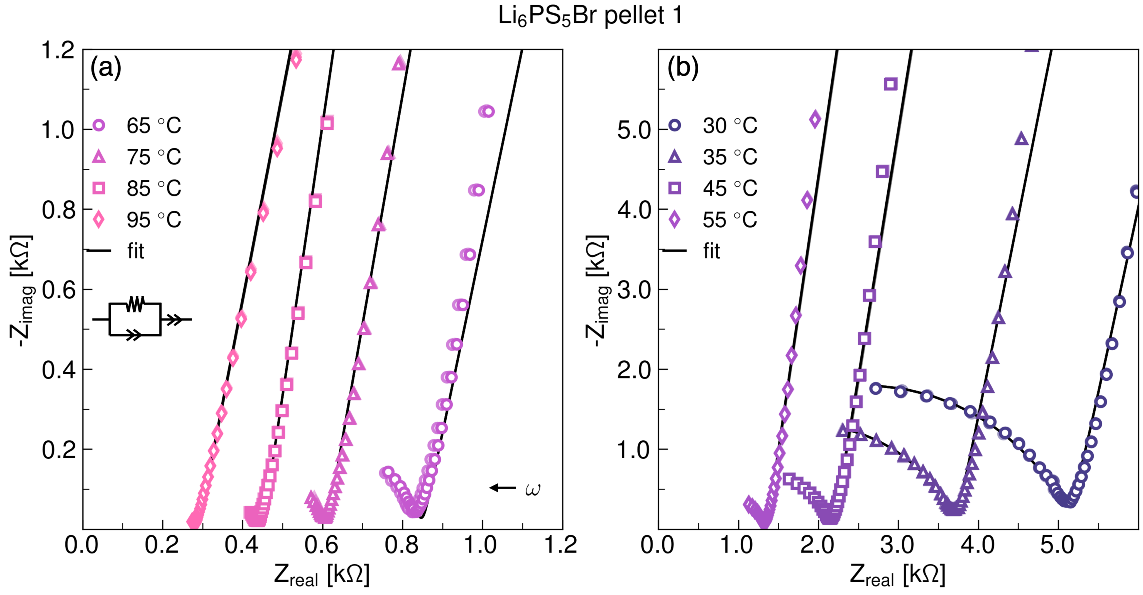

Figure S18: Temperature-dependent Nyquist plots of the last 3 replicate scans (decreasing transparency with later replicates) at each temperature for pellet 1 of Li_6PS_5Br. (a) High and (b) low temperature data have been separated for clarity. Data points are represented by markers, and the solid lines indicate the calculated fit to the $(R_1Q_1) + Q_2$ equivalent circuit model.

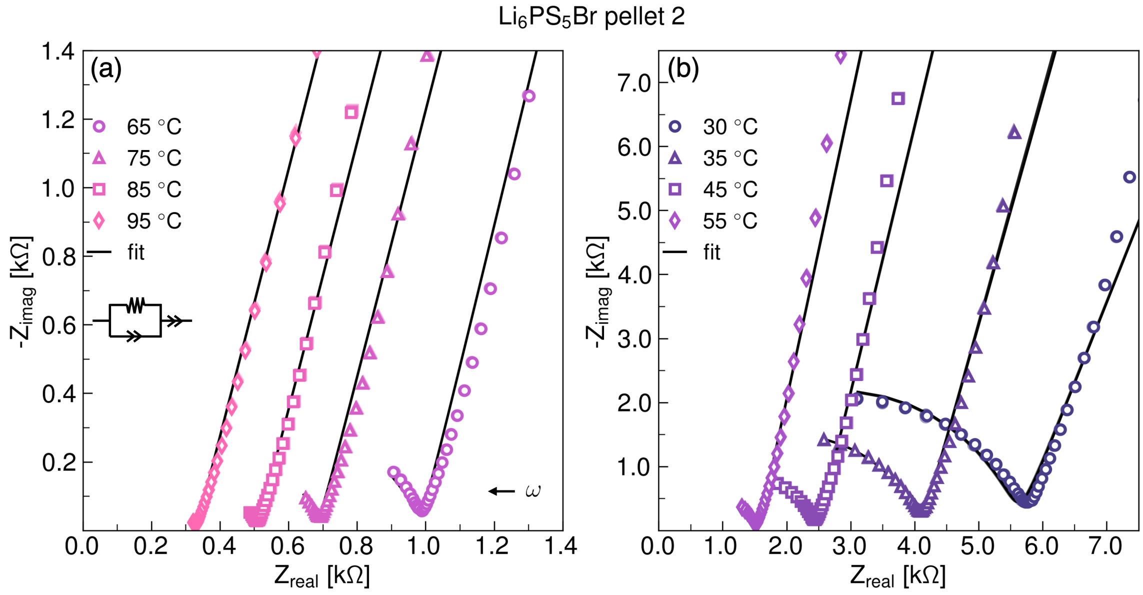

Figure S19: Temperature-dependent Nyquist plots of the last 3 replicate scans (decreasing transparency with later replicates) at each temperature for pellet 2 of Li_6PS_5Br. (a) High and (b) low temperature data have been separated for clarity. Data points are represented by markers, and the solid lines indicate the calculated fit to the $(R_1Q_1) + Q_2$ equivalent circuit model.

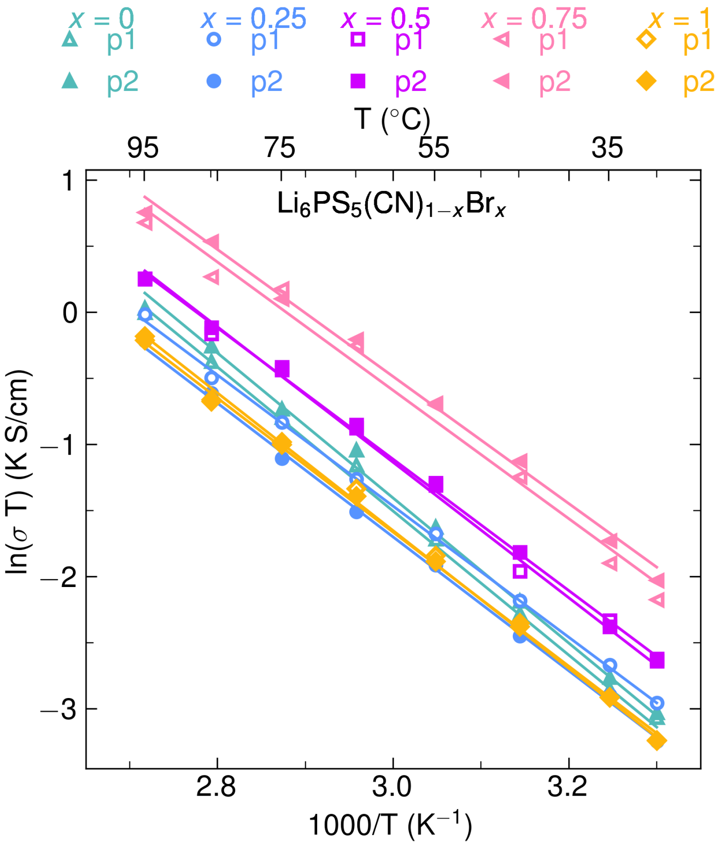

Figure S20: Arrhenius relationships for 2 pellets for each x across the $Li_6PS_5(CN)_{1-x}Br_x$ series determined from temperature-dependent electrochemical impedance spectroscopy. The linear regression is shown as a solid line. Error bars represent the standard deviation between replicate scans at each temperature, and are generally smaller than the markers.

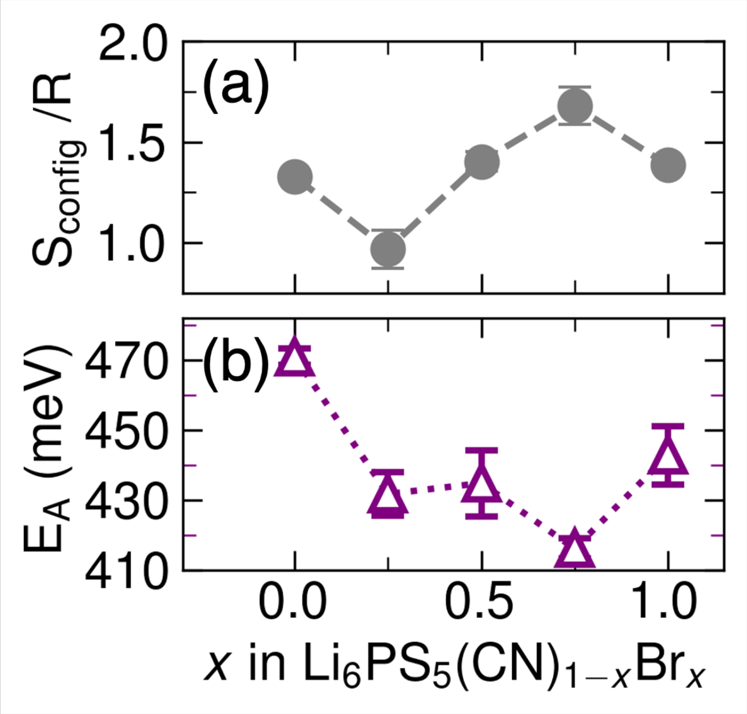

Figure S21: (a) Configurational entropy (S_{config}) normalized by the ideal gas constant reaches a maximum for the composition x that minimizes the (b) activation energy (E_A) in $Li_6PS_5(CN)_{1-x}Br_x$. Error bars for S_{config} represent error for anion occupancies on 4a and 4d propagated through equation 1. Error bars for E_A show the standard deviation between E_A values extracted from the Arrhenius fit to temperature-dependent electrochemical impedance spectroscopy measurements between two pellets.

Heat Capacity

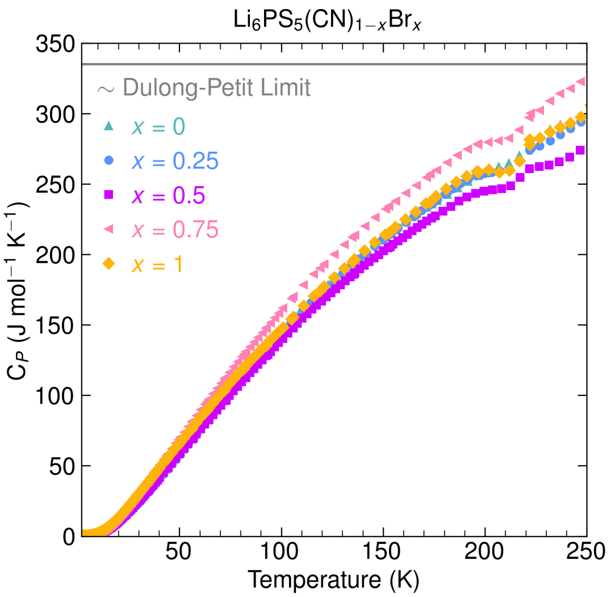

Figure S22: Heat capacity data collected from 2 to 250 K for $Li_6PS_5(CN)_{1-x}Br_x$. We note that the feature near ∼230 K is an artifact of the instrument, as it is observed across a wide range of samples.

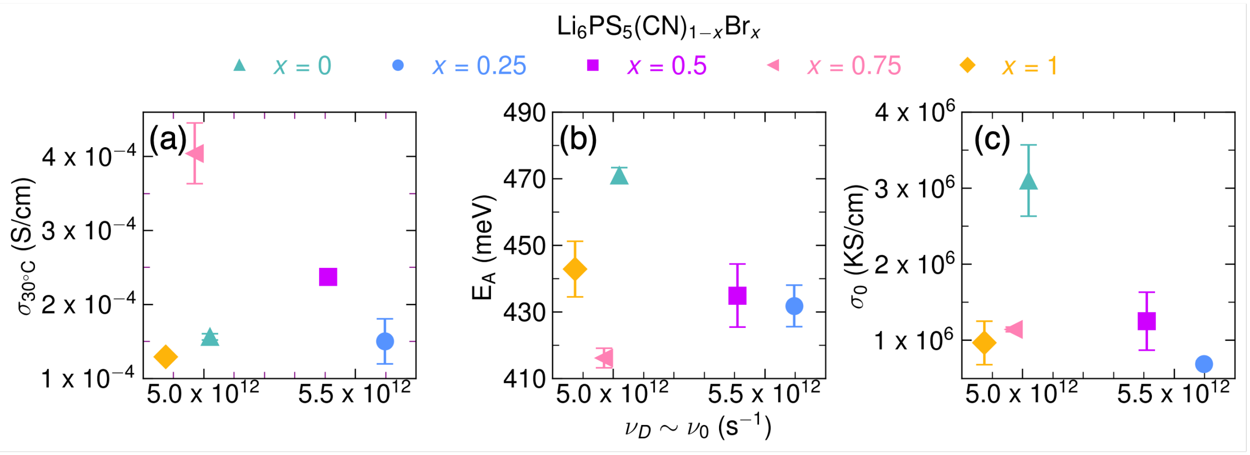

Figure S23: (a) Ionic conductivity at 30 °C ($\sigma_{30°C}$), (b) activation energy (E_A), and (c) the Arrhenius prefactor to the conductivity (σ_0) as functions of Debye frequency (ν_D) extracted from the Debye model fits to the low temperature heat capacity data in $Li_6PS_5(CN)_{1-x}Br_x$. Error bars for $\sigma_{30°C}$, σ_0, and E_A show the standard deviation between two pellets.

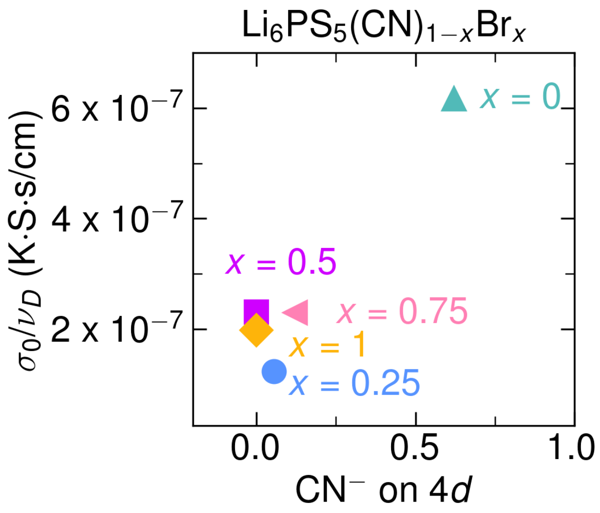

Figure S24: Arrhenius prefactor normalized by the Debye frequency (σ_0/ν_D, KSs/cm) as a function of CN^- occupancy on 4*d* in $Li_6PS_5(CN)_{1-x}Br_x$.

References

(1) Loidl, A.; Schräder, T.; Knorr, K.; Böhmer, R.; Mertz, B.; McIntyre, G.; Vogt, T.; Mutka, H.; Müllner, M.; Jex, H.; others Elastic and quasielastic neutron scattering studies in KBr:KCN mixed crystals. *Z. Phys. B* **1989**, *75*, 81–99.

(2) Field, G. R.; Sherman, W. F. Cyanide ion - Environmental perturbation of its vibrational and rotational motion when isolated in alkali halides. *J. Chem. Phys.* **1967**, *47*, 2378–2389.

(3) Trzebiatowska, M. The spectroscopic study of phase transitions in the series of cyanide perovskites. *Spectrochim Acta A Mol Biomol Spectrosc.* **2021**, *245*.

(4) Roedel, E.; Urakawa, A.; Kureti, S.; Baiker, A. On the local sensitivity of different IR techniques: Ba species relevant in NO_x storage-reduction. *Phys. Chem. Chem. Phys.* **2008**, *10*, 6190–6198.

(5) Dietrich, C.; Weber, D. A.; Culver, S.; Senyshyn, A.; Sedlmaier, S. J.; Indris, S.; Janek, J.; Zeier, W. G. Synthesis, Structural Characterization, and Lithium Ion Conductivity of the Lithium Thiophosphate $Li_2P_2S_6$. *Inorg. Chem.* **2017**, *56*, 6681–6687.

(6) Schlem, R.; Ghidiu, M.; Culver, S. P.; Hansen, A.-L.; Zeier, W. G. Changing the static and dynamic lattice effects for the improvement of the ionic transport properties within the argyrodite $Li_6PS_{5-x}Se_xI$. *ACS Appl. Energy Mater.* **2020**, *3*, 9–18.

(7) Sakuda, A.; Yamauchi, A.; Yubuchi, S.; Kitamura, N.; Idemoto, Y.; Hayashi, A.; Tatsumisago, M. Mechanochemically Prepared Li_2S-P_2S_5-$LiBH_4$ Solid Electrolytes with an Argyrodite Structure. *ACS Omega* **2018**, *3*, 5453–5458.

(8) Suto, K.; Bonnick, P.; Nagai, E.; Niitani, K.; Arthur, T. S.; Muldoon, J. Microwave-aided synthesis of lithium thiophosphate solid electrolyte. *J. Mater. Chem. A* **2018**, *6*, 21261–21265.

(9) Ito, S.; Nakakita, M.; Aihara, Y.; Uehara, T.; Machida, N. A synthesis of crystalline $Li_7P_3S_{11}$ solid electrolyte from 1,2-dimethoxyethane solvent. *J. Power Sources* **2014**, *271*, 342–345.

(10) Sadowski, M.; Albe, K. Influence of Br^-/S^{2-} site-exchange on Li diffusion mechanism in Li_6PS_5Br: A computational study. *Philos. Trans. R. Soc. A* **2021**, *379*, 20190458.

(11) Fuchs, T.; Culver, S. P.; Till, P.; Zeier, W. G. Defect-Mediated Conductivity Enhancements in $Na_{3-x}Pn_{1-x}W_xS_4$ (Pn = P, Sb) Using Aliovalent Substitutions. *ACS Energy Lett.* **2020**, *5*, 146–151.

(12) Yubuchi, S.; Uematsu, M.; Hotehama, C.; Sakuda, A.; Hayashi, A.; Tatsumisago, M. An argyrodite sulfide-based superionic conductor synthesized by a liquid-phase technique with tetrahydrofuran and ethanol. *J. Mater. Chem. A* **2019**, *7*, 558–566.

(13) Gautam, A.; Sadowski, M.; Prinz, N.; Eickhoff, H.; Minafra, N.; Ghidiu, M.; Culver, S. P.; Albe, K.; Fässler, T. F.; Zobel, M.; Zeier, W. G. Rapid Crystallization and Kinetic Freezing of Site-Disorder in the Lithium Superionic Argyrodite Li_6PS_5Br. *Chem. Mater.* **2019**, *31*, 10178–10185.
